# Poly I:C Activated Microglia Disrupt Perineuronal Nets and Modulate Synaptic Balance in Primary Hippocampal Neurons *in vitro*

**DOI:** 10.3389/fnsyn.2021.637549

**Published:** 2021-02-23

**Authors:** David Wegrzyn, Nadja Freund, Andreas Faissner, Georg Juckel

**Affiliations:** ^1^Department of Cell Morphology and Molecular Neurobiology, Ruhr-University Bochum, Bochum, Germany; ^2^Division of Experimental and Molecular Psychiatry, Department of Psychiatry, Psychotherapy and Preventive Medicine, LWL University Hospital, Ruhr-University Bochum, Bochum, Germany

**Keywords:** aggrecan, cytokines, microglia, multielectrode array, perineuronal nets, Poly I:C, schizophrenia, synaptic plasticitiy

## Abstract

Perineuronal nets (PNNs) are specialized, reticular structures of the extracellular matrix (ECM) that can be found covering the soma and proximal dendrites of a neuronal subpopulation. Recent studies have shown that PNNs can highly influence synaptic plasticity and are disrupted in different neuropsychiatric disorders like schizophrenia. Interestingly, there is a growing evidence that microglia can promote the loss of PNNs and contribute to neuropsychiatric disorders. Based on this knowledge, we analyzed the impact of activated microglia on hippocampal neuronal networks *in vitro*. Therefore, primary cortical microglia were cultured and stimulated via polyinosinic-polycytidylic acid (Poly I:C; 50 μg/ml) administration. The Poly I:C treatment induced the expression and secretion of different cytokines belonging to the CCL- and CXCL-motif chemokine family as well as interleukin-6 (IL-6) and tumor necrosis factor-α (TNF-α). In addition, the expression of matrix metalloproteinases (MMPs) could be verified via RT-PCR analysis. Embryonic hippocampal neurons were then cultured for 12 days *in vitro* (DIV) and treated for 24 h with microglial conditioned medium. Interestingly, immunocytochemical staining of the PNN component Aggrecan revealed a clear disruption of PNNs accompanied by a significant increase of glutamatergic and a decrease of γ-aminobutyric acid-(GABA)ergic synapse numbers on PNN wearing neurons. In contrast, PNN negative neurons showed a significant reduction in both, glutamatergic and GABAergic synapses. Electrophysiological recordings were performed via multielectrode array (MEA) technology and unraveled a significantly increased spontaneous network activity that sustained also 24 and 48 h after the administration of microglia conditioned medium. Taken together, we could observe a strong impact of microglial secreted factors on PNN integrity, synaptic plasticity and electrophysiological properties of cultured neurons. Our observations might enhance the understanding of neuron-microglia interactions considering the ECM.

## Introduction

Microglia, the immune cells of the central nervous system (CNS), were first described by Pio del Rio-Hortega more than a century ago (del Rio-Hortega, 1919, del Rio-Hortega, 1932). Their distribution in the CNS is not uniform and they make up a proportion of 5–12% depending on the brain region (Lawson et al., 1990). Interestingly, a higher density of microglia could be observed in the hippocampus, the basal ganglia and the substantia nigra (Lawson et al., 1990; Tan et al., 2019). The origin of microglia was unknown for a long time in this field of research (Ginhoux and Garel, 2018). However, there is a growing evidence that microglia originate during early development from progenitors in the extra-embryonic yolk sac, migrate into the developing CNS and persist until adulthood (Alliot et al., 1999; Ginhoux et al., 2013; Ginhoux and Garel, 2018). Through the release of cytokines, reactive oxygen species (ROS) and their phagocytic ability, microglia fulfill an essential function for the defense of the CNS and contribute to the maintenance of CNS homeostasis (Casano and Peri, 2015). In addition, several studies revealed that microglia can modulate synapse numbers and are involved in synaptic maturation as well as synaptic pruning (Paolicelli et al., 2011; Schafer et al., 2012). Weinhardt and colleagues demonstrated that microglia engulf presynaptic structures by a process called trogocytosis and modulate dendritic spine morphology (Weinhard et al., 2018). Surprisingly, it has been unraveled that neurons express many functional cytokine receptors and respond to the release of microglial immune factors (Viviani et al., 2003; Stellwagen et al., 2005; Gardoni et al., 2011; Vezzani and Viviani, 2015). On the other hand, neurons can modify the behavior of microglia. Microglia express receptors for different neuronal neurotransmitters like glutamate, GABA or serotonin (Pocock and Kettenmann, 2007; York et al., 2018). In this way microglia and neurons influence each other in a bidirectional way (Szepesi et al., 2018).

Less is known about the impact of microglia on PNNs, however a recently published study revealed that microglia can facilitate the loss of PNNs in an Alzheimer's disease model (Crapser et al., 2020). PNNs are dense mesh-like structures and consist of a heterogeneous composition of ECM molecules, which are covering the soma and proximal dendrites of neurons. They were first described by Camillo Golgi in 1898 and are often associated with fast spiking, parvalbumin positive interneurons (Brückner et al., 1994; Härtig et al., 1994; Celio et al., 1998; Carulli et al., 2010; Fawcett et al., 2019). Since their discovery, many studies exposed a multifunctional role of PNNs including the restriction of synaptic plasticity (Pizzorusso et al., 2002; Gogolla et al., 2009; Carstens et al., 2016; Lensjø et al., 2017), maturation of interneurons by Otx2 binding (Beurdeley et al., 2012), neuroprotection (Morawski et al., 2004; Cabungcal et al., 2013), ion sorting and buffering (Härtig et al., 1999; Morawski et al., 2015), receptor stabilization and motility (Frischknecht et al., 2009) as well as the maintenance of an excitatory/inhibitory balance (Gottschling et al., 2019). Evidence is mounting that PNNs are impaired in schizophrenia patients and animal models for schizophrenia (Berretta, 2012). Especially *post-mortem* studies revealed a significant reduction of PNN density and staining intensity in schizophrenia patients (Pantazopoulos et al., 2010; Mauney et al., 2013; Bitanihirwe and Woo, 2014; Enwright et al., 2016). Similar observations were made in a prenatal maternal immune activation animal model for schizophrenia (Paylor et al., 2016). While many studies describe the impairments of PNNs, the exact cause of this alteration is not known. Interestingly, an elevated number of activated microglia and an increased inflammatory response were observed in schizophrenia patients or animal models (Bayer et al., 1999; Radewicz et al., 2000; Juckel et al., 2011; Fillman et al., 2013). In addition, patient derived microglia-like cells showed a higher synapse engulfment and elimination *in vitro* (Sellgren et al., 2019). Furthermore, microglia can release a variety of cytokines and matrix metalloproteinases, including MMP-2 and MMP-9 (Könnecke and Bechmann, 2013). Both possess the ability to induce a direct or indirect cleavage of aggrecan or brevican which are strongly expressed in PNNs (Overall, 2002; Ethell and Ethell, 2007). Therefore, we suppose that microglial secreted factors might influence the integrity of PNNs and synaptic plasticity.

In the following study, we used an indirect co-culture system of hippocampal neurons and cortical astrocytes in order to culture neuronal networks in serum-free, completely defined medium (Geissler and Faissner, 2012; Gottschling et al., 2016). After a culturing time of 12 days we removed the astrocyte-containing inserts and partly exchanged the neuronal medium by conditioned medium of Poly I:C activated microglia. Using this setup, we analyzed the impact of microglia secreted factors on PNNs, structural synapse numbers and electrophysiological network properties. Our experiments are limited to the activated state of microglia and focus on their influence on the above-mentioned parameters. Inactive microglia were not included since a relative high percentage of active microglia was observed in the vehicle control condition (0 μg/ml Poly I:C), which was presumably induced by primary culturing procedures. A better understanding of neuron-microglia interaction might help to develop supporting therapies for different neurological and neuropsychiatric diseases in the future.

## Materials and Methods

### Animal Housing and Ethical Standards

For all experiments SV129 wild-type mice of both sexes were used in accordance with the Society for Neuroscience and the European Council Directive of September 22, 2010 (2010/63/EU), for care of laboratory animals and approved by the animal care committee of North Rhine-Westphalia, Germany, based at the LANUV (Landesamt für Umweltschutz, Naturschutz und Verbraucherschutz, North Rhine-Westphalia, D-45659 Recklinghausen, Germany). The animals were kept under standardized conditions in conventional housing with regulated humidity (60–70%), temperature (21°C), and 12 h dark/light circle as well as food and water supply *ad libitum*. Animals originated from the mouse colony of the Department of Cell Morphology and Molecular Neurobiology of the Ruhr-University Bochum.

### Cell Culture

#### Mixed Glial Culture

The preparation of mixed glial cultures was performed as previously described, with minor modifications (McCarthy and de Vellis, 1980; Gottschling et al., 2016). Briefly, six cortices of postnatal day 1–3 (P1–3) mice were isolated and digested with 30 U Papain [Worthington; Cat. No.: LS003126)], 80 μg/ml (w/v) DNase I (Worthington; Cat. No.: LS002007), and 0.96 mg/ml (w/v) L-cysteine (Sigma-Aldrich; Cat. No.: C2529) in DMEM (Thermo Fisher Scientific Inc.; Cat. No.: 41966029) for 1 h at 37°C. Next, digestion solution was replaced by astrocyte medium [DMEM (Thermo Fisher Scientific Inc.: Cat. No.: 41966029) with 10% (v/v) horse serum (Sigma-Aldrich; Cat. No.: S9135) and 0.1% (v/v) gentamicin (Sigma-Aldrich; Cat. No.: S9135)]. Afterwards, cortical tissue was carefully triturated to a single-cell suspension and subsequently centrifuged at 1,000 rpm for 5 min. After discarding the supernatant, the remaining cell pellet was re-suspended in 1 ml fresh astrocyte medium. Finally, the re-suspended cell solution was added to 9 ml astrocyte medium in a T-75 culture flask (Sarstedt; Cat. No.: 83.3911.002). The culture flask was previously coated with 10 μg/ml (w/v) poly-D-lysine (PDL, Sigma-Aldrich; Cat. No.: P0899) for 1 h at 37°C. The mixed glial cells were cultured at 37°C and 6% CO_2_ with a first total medium exchange after 7 days. Then, total medium exchanges were performed every second day.

#### Culturing of Pure Astrocytes

After 7–8 days astrocytes have formed a dense monolayer with microglia and oligodendrocyte precursor cells (OPCs) on the top. To maintain a pure astrocyte culture, T-75 flasks were pre-shaken on an orbital shaker for 1 h at 250 rpm and 37°C (New Brunswick Scientific). Then, the medium was completely exchanged, and the T-75 flasks were incubated for 1 h at 37°C and 6% CO_2_ for equilibration. Afterwards, the culture flasks were shaken overnight at 250 rpm and 37°C. Previously, the lid of each culture flask was covered with parafilm (Brand, Wertheim) to sustain the CO_2_ content in the flask. Next day, the medium was completely exchanged and 10 μl (≙ 20 mM) of cytosine-1-β-D- arabinofuranoside (Ara-C, Sigma Aldrich, Cat. No.: C1768) were supplied for 48–72 h. Because of its cytotoxic effect on mitotic cells, Ara-C reduced the amount of microglia and oligodendrocyte precursor cells. After the Ara-C treatment normal medium exchange was performed every second day, as previously described.

For the neuron-astrocyte co-cultures, pure astrocytes were plated in cell culture transwells with a permeable membrane (pore size: 0.4 μm; Falcon by Thermo Fisher Scientific Inc; Cat. No. 08-770), that allows for the exchange of released factors into the commonly shared medium (Geissler und Faissner 2012). First, astrocytes were incubated with 0.05 % (v/v) trypsin/EDTA (T/E) (Thermo Fisher Scientific Inc.: Cat. No.: 25300054) for 5–7 min at 37°C and 6% CO_2_. The detached astrocytes were added to 7 ml of astrocyte medium and centrifuged at 1,000 rpm for 5 min. Afterwards, the supernatant was removed, and the astrocyte pellet re-suspended in 1 ml fresh medium. After determining the cell number, 25.000 astrocytes were plated out on PDL-coated inserts and cultured at 37°C and 6% CO_2_ with a regular medium exchange every second day. After ~4–5 days astrocytes were confluent, covering the membrane of the transwell and could be used for the co-cultures.

#### Culturing of Primary Microglia

For the culturing of primary microglia, cortical mixed glial cultures were prepared, as previously described in Mixed Glial Culture. Instead of shaking the culture flasks after observing an astrocytic confluence, the medium was not changed for 5 days, to enrich the number of microglia. After 5 days, culture flasks were shaken at 200 rpm on an orbital shaker for 1 h. Then, the supernatant of the T-75 flasks was centrifuged at 1,000 rpm for 5 min. The supernatant was discarded, and the pellet resuspended in microglia medium consisting of DMEM (Thermo Fisher Scientific Inc.: Cat. No.: 41966029) with 10 % (v/v) FCS (Sigma-Aldrich; Cat. No.: F6765), 1 % (v/v) sodium pyruvate (Sigma-Aldrich; Cat. No.: S8636) and 0.1 % (v/v) gentamicin (Sigma-Aldrich; Cat. No.: S9135). Because of the low cell number, microglia of two T-75 flasks were put together in one approach. Finally, the number of microglial cells was determined and plated out in a defined density.

#### Microglial Activation Assay

For the morphological analysis of microglia after the addition of increasing Poly I:C-concentrations, 20.000 microglia in 500 μl medium (≙ 40.000 cells/ml) were plated out per well. Before, glass coverslips were placed in 4-well plates (Thermo Fisher Scientific Inc.: Cat. No.: 176740) and coated with 10 μg/ml (w/v) PDL (Sigma-Aldrich; Cat. No.: P0899) for 1 h at 37°C in the incubator. After culturing the microglia for 2 days at 37°C and 6% CO_2_, half of the medium was removed and replaced by fresh microglia medium or by microglia medium containing 50 or 100 μg/ml Poly I:C (Sigma Aldrich; Cat. No.: P1530). Subsequent, the 4-well plates were placed in the incubator for 3 h. The incubation time and Poly I:C concentrations used in this study were adapted from a previous publication which showed that Poly I:C activates microglia *in vitro* by toll-like receptor 3 (TLR-3) signaling (Town et al., 2006). Then, microglia were fixed with pre-warmed 4% (w/v) paraformaldehyde (Carl Roth GmbH & Co. KG; Cat. No.: 4235.1) for 10 min. After three washing steps with PBS (1x) the cells were stored at 4°C until immunocytochemical staining were performed.

#### Conditioning of Hippocampus Medium

First, T-25 culture flasks (Sarstedt; Cat. No.: 83.3919.002) were coated with 10 μg/ml PDL (Sigma-Aldrich; Cat. No.: P0899). Next, 200.000 microglia in 2 ml microglia medium (≙ 100.000 cells/ml) were plated out and cultured at 37°C and 6% CO_2_. After 2 days *in vitro*, the microglia medium was completely removed, and the microglia were washed once with PBS (1x). Afterwards, 1 ml hippocampus medium with 50 μg/ml Poly I:C was added to the culture flasks. After an additional culturing time of 24 h, the medium was collected and stored at −21°C for further analysis. Media collection was performed after 24 h similar to the aforementioned study design by Town and colleagues who observed a higher enrichment of cytokines in the supernatant of Poly I:C activated microglia after 24 h in comparison to an earlier collection after 8 h.

#### Culturing of Primary Hippocampal Neurons

The culturing of primary hippocampal neurons was performed, as previously described (Gottschling et al., 2016; Wegrzyn et al., 2020). The hippocampi of embryonic stage 15.5 (E15.5) mice were isolated and collected in 1 ml dissection medium [(HBSS, Thermo Fisher Scientific Inc.; Cat. No.: 14170088), 0.6% (w/v) glucose (Serva Electrophoresis GmbH; Cat. No.: 22700), and 10 mM HEPES (Thermo Fisher Scientific Inc; Cat. No.: 15630080)]. After removing the dissection medium, tissue was digested using 30 U Papain (Worthington; Cat. No.: LS003126) in MEM (Thermo Fisher Scientific Inc; Cat. No.: 31095029) with 80 μg/ml (w/v) DNase I (Worthington; Cat. No.: LS002007) and 0.96 mg/ml (w/v) L-cysteine (Sigma-Aldrich; Cat. No.: C2529). The digestion was carried out at 37°C for 15 min. Afterwards, the digestion solution was removed, and the hippocampi were washed three times with freshly prepared hippocampus medium [MEM (Thermo Fisher Scientific Inc; Cat. No.: 31095029), 2% (v/v) B27 (Thermo Fisher Scientific Inc; Cat. No.: 17504044), 0.1% (v/v) ovalbumin (Sigma-Aldrich; Cat. No.: A7641) 10 mM sodium pyruvate (Sigma-Aldrich; Cat. No.: S8636), and 0.1% (v/v) gentamicin (Sigma-Aldrich; Cat. No.: S9135)]. After the last washing step, the hippocampi were gently triturated to a single-cell suspension and the cell number was determined. Finally, a dilution with a defined cell number was prepared and used for the experiments.

#### Neuron Astrocyte Co-culture and Treatment

Primary hippocampal neurons were prepared as described in Culturing of Primary Hippocampal Neurons. First glass cover slips were coated with poly-L-ornithine (PLO) (15 μg/ml; Sigma-Aldrich; Cat. No.: P3655) for 1 h at 37°C. Here, special 24-well plates were used (Falcon; Product No.: 353504) because of the later addition of astrocyte containing transwells. After determining the neuronal cell number, 35.000 neurons were plated out in 500 μl hippocampus medium per well (≙ 70.000 cells/ml). The 24-well plates were placed in the incubator for 1 h at 37°C and 6% CO_2_. In this time, the astrocyte medium in the previously prepared transwells with astrocytes, was replaced by hippocampus medium. After the neurons had settled down, inserts with astrocytes were carefully hung in the 24-well plates. Finally, co-cultures were placed in the incubator and cultured at 37°C and 6% CO_2_. After a culturing time of 12 days, the inserts with astrocytes were removed and discarded because astrocytes express toll-like receptors and might falsify results (Bowman et al., 2003). Then a 25% v/v medium exchange was performed, and the microglial conditioned medium was carefully added to the neuronal cultures. After an incubation time of 24 h neuronal cultures were fixed with pre-warmed 4% (w/v) paraformaldehyde. Then, cultures were washed three times with PBS (1x) and stored at 4°C until further experiments were performed.

### Electrophysiology

#### Astrocyte-Neuron Co-culture on MEAs

For the analysis of the spontaneous network activity of cultured hippocampal neurons after adding microglia conditioned medium, multielectrode array analysis was performed (Geissler and Faissner, 2012). The following standard MEAs were used for the experiments: 60MEA200/30iR-Ti-gr (Multichannel systems; Item No.: 890103). First, electrode fields of MEAs were coated with 0.05% (v/v) poly-ethylenimine (PEI, Sigma-Aldrich; Cat. No.: P3143) for 1 h at room temperature. Then, electrode fields were washed 4–5 times with sterile Milli-Q water (Millipore) and air dried under UV-light for a minimum of 30 min. Next, 30 μl of a laminin solution (5 μg/ml (w/v), Corning; Cat. No.: 354259) were placed directly on the electrode field and incubated for 20 min at 37°C and 6% CO_2_. After this secondary coating, the laminin solution was aspirated and 30.000 neurons in 30 μl hippocampus medium (≙ 1 × 10^6^ cells per ml) were subsequently placed on the electrode field, without any washing step. After a visual control of the cell density via phase contrast microscopy, an additional volume of 30 μl cell suspension was added if it was necessary. The MEAs were incubated for 10 min at 37°C and 6% CO_2_ in the incubator and afterwards flooded with 1 ml hippocampus medium. After an incubation time of 1 h in the incubator, inserts with confluently grown astrocytes were placed in specially constructed racks and added to the MEA cultures. Before, the astrocyte medium was replaced by hippocampus medium. Next, the co-cultures were placed in plastic boxes with a fluorinated ethylene-propylene lid, which allows for a gas exchange in the incubator but reduces the evaporation.

#### Medium Exchange Experiments and Data Quantification

For the measurement of the spontaneous network activity after a partial medium exchange with microglia conditioned medium, neurons were cultured for 12 days under the previously mentioned conditions. Astrocytes are expressing toll-like receptors and might react to either the Poly I:C or the microglia secreted factors in the conditioned medium (Bowman et al., 2003). Therefore, inserts with astrocytes were removed and not used for the further experimental course after 12 DIV. After removing the astrocytes, MEAs were placed on a 35°C pre-heated pre-amplifier for 10 minutes. Then, baseline recordings were performed for 10 minutes using a sampling frequency of 20 kHz. The program MCRack (Version 3.9.0, Multichannel Systems) was used for the data recording. A high-pass filter was adjusted at a frequency of 200 Hz to eliminate field potentials from the raw data. Furthermore, a spike detector recorded single spontaneous amplitudes 4.5-fold higher than the standard deviation. For the detection of bursts following parameters were used: maximal interval initiating a burst: 10 ms, maximal interval terminating a burst: 100 ms, maximal interval between two bursts: 210 ms, minimal duration of a burst: 50 ms, minimal number of spikes per burst: 5.

After the baseline recording, a 25% v/v medium exchange was performed. Here, three different media were used: conditioned hippocampus medium from 24 h activated microglia with Poly I:C (50 μg/ml), hippocampus medium containing Poly I:C (50 μg/ml) and hippocampus medium only. After the medium exchange was performed, MEAs were placed in the aforementioned plastic boxes and transferred to the incubator. Next, MEA recordings were performed 3, 24, and 48 h after the medium exchange, using the previously mentioned setting. At the end of the experiment, the media were aspirated and discarded. Then, 1% (w/v) tergazyme (Alconox; Cat. No.: 1304) was added to the MEAs and incubated overnight. Next day, the tergazyme solution was removed together with all cell debris and MEAs were subsequently washed three times with Milli-Q water (Millipore). Finally, MEAs were stored at 4°C until next usage. For the quantification in Figures 4D–F the absolute number of spikes detected by every single electrode during the baseline measurement was exported and sorted based on the electrode number. Then, the absolute number of spikes recorded by every single electrode after 3, 24, and 48 h of treatment was exported and arranged to its corresponding electrode of the baseline measurement. In this way, the absolute increase or decrease of spikes detected by every single electrode could be calculated by subtraction (e.g., when electrode number 55 detected 1,500 spikes in the baseline measurement and 1,700 spikes after 3 h of treatment an absolute increase of 200 spikes was noticed). Then, the mean of all absolute increase/decrease values was calculated and presented in Figure 4. The same procedure was performed for all other parameters in Figures 4D–F.

### Immunocytochemistry

#### Visualization of Apoptotic Microglia via Iba1/Caspase-3 Staining

For the identification of microglia an immunocytochemical staining against ionized calcium-binding adapter molecule 1 (Iba1) was performed. In order to exclude that a rounding of microglia after a 3 h Poly I:C exposure is a pure apoptosis effect, we concomitantly performed the caspase evaluation at this point in time. First, microglia were incubated with pre-warmed 4% (w/v) paraformaldehyde (Carl Roth GmbH & Co. KG; Cat. No.:4235.1) for 10 min. Afterwards, the paraformaldehyde solution was removed, and the microglia were washed three times with PBS (1x). Next, primary antibodies were diluted in PBT-1 (PBS with 0.1% (v/v) Triton X-100; (AppliChem GmbH; Cat. No.: A4975,0500) and 1% (w/v) BSA (Carl Roth GmbH & Co. KG; Cat. No.:8076.2). Here, a polyclonal anti-Iba1 antibody (1:300, goat, Abcam; Cat. No.: ab5076, RRID:AB_2224402) and an antibody against active Caspase 3 (1:300; rabbit; polyclonal, Sigma-Aldrich; RRID:AB_476884) was used. The microglia were incubated 1 h with primary antibody solution at room temperature. After removing the primary antibody solution, coverslips were rinsed three times with PBS/A (PBS containing 0.1% (w/v) BSA; Carl Roth GmbH & Co. KG; Cat. No.:8076.2). As secondary antibodies a polyclonal anti-rabbit [1:500; Cy3; IgG (H+L); Jackson Immuno Research; RRID: AB_2338003)] and a polyclonal donkey anti-goat (1:250; AF488; IgG (H+L); Jackson Immuno Research; RRID: AB_ AB_2336933) antibody were used. In addition, bisbenzimide (Hoechst 33258, 1:100.000, Sigma-Aldrich) was added to the secondary antibody solution for the visualization of nuclei. Coverslips were incubated for 45–60 min with the secondary antibody solution at room temperature. Then, the coverslips were washed three times with PBS (1x), once with Milli-Q water and finally covered on microscope slides (Thermo Scientific; Cat. No.: 630-2012) with ImmuMount (Thermo Scientific; Cat. No.: 10662815). The microscope slides were stored at 4°C until fluorescence microscopy was performed.

#### Visualization of Glutamatergic Synapses

For the triple staining of glutamatergic synapses on PNN positive neurons, coverslips were washed three times with washing solution containing 10% (v/v) fetal bovine serum (FBS) (Sigma-Aldrich; Cat. No.: F7524), 0.1 mM glycine (VWR International GmbH; Cat. No.: 101196X), and 0.1% (v/v) Triton X-100 (AppliChem; Cat. No.: A4975,0500) in PBS, after fixation. Then, following antibodies were diluted in washing solution: anti-vesicular glutamate transporter-1(vGlut) (1:1,000; guinea pig; polyclonal; Synaptic Systems GmbH; RRID: AB_887878), anti-postsynaptic density-95 (PSD-95) (1:500; mouse; monoclonal IgG2a; Merck KGaA; RRID: AB_1121285), and anti-aggrecan (1:500; rabbit; polyclonal; Merck KGaA; RRID: 90460). Coverslips were incubated for 1 h at room temperature. Then, coverslips were washed three times with washing solution and incubated with the secondary antibody solution for 1 h at room temperature. Here, following antibodies were used: anti-guinea pig (1:500; AF647; IgG (H+L); Jackson Immuno Research, RRID: AB_2337446), anti-mouse, (1:250; AF488; IgM+IgG (H+L); Jackson Immuno Research; RRID: AB_2338844), and anti-rabbit (1:500; Cy3; IgG (H+L); Jackson Immuno Research; RRID: AB_2338003). Last, coverslips were washed three times with PBS (1x), once with MilliQ water and finally mounted on microscope slides.

#### Visualization of GABAergic Synapses

The visualization of inhibitory synaptic proteins on PNN wearing neurons was performed using a staining protocol by Dobie and colleagues (Dobie and Craig, 2011). Briefly, neurons were fixed for 10 min with pre-warmed 4% (w/v) paraformaldehyde (Carl Roth GmbH & Co. KG; Cat. No.:4235.1). Afterwards, neurons were washed three times with 0.25% (v/v) Triton X-100 (AppliChem GmbH; Cat. No.: A4975,0500) in PBS and blocked with 10% (w/v) BSA (Carl Roth GmbH & Co. KG; Cat. No.:8076.2) for 30 min at 37°C. Then, primary antibodies were diluted in 3% (w/v) BSA in PBS and incubated for 2 h at 37°C. Following antibodies were used for the triple staining: anti-vesicular GABA transporter (vGAT) (1:500; guinea pig; polyclonal; Synaptic Systems GmbH; RRID: AB_887873), anti-gephyrin 1:500; mouse; monoclonal Ig1; Synaptic Systems GmbH; RRID: AB_887717), and anti-aggrecan (1:500; rabbit; polyclonal; Merck KGaA; RRID: 90460). After three washing steps with 3% (w/v) BSA in PBS, secondary antibodies were diluted and incubated for 1 h at 37°C. Here, same secondary antibodies were used as previously mentioned in section Visualization of Glutamatergic Synapses. Finally, coverslips were washed twice with PBS, once with MilliQ water and finally mounted on microscope slides.

### Molecular Biology

#### mRNA Isolation

For the mRNA isolation of cultured cortical astrocytes, the GenElute Mammalian Total RNA Miniprep Kit (Sigma-Aldrich by Merck KGaA; Cat. No.: RTN350) was utilized after collecting the conditioned medium. The procedure was performed without changes concerning the manufacturer's protocol.

#### cDNA Synthesis

The cDNA synthesis was performed utilizing the First *Strand cDNA Synthesis Kit* from Thermo Fisher Scientific (Cat. No.: K1622). Due to the low amount of mRNA isolated from cultured microglia, 40 μL of lysates were used for the cDNA synthesis. No additional changes were performed concerning the manufacturer's protocol.

#### RT-PCR Analysis

Primers were obtained from Sigma-Aldrich. The detailed primers sequences and product lengths can be found in Table 1.

**Table 1:** Primer sequences for PCR analysis.

**Gene**	**Primer sequence**	**Product length**
*IL1b for*	5′ TGCCACCTTTTGACAGTGATG 3′	555 bp
*IL1b rev*	5′ TGGGTGTGCCGTCTTTCATT 3′	
*IL6 for*	5′ GCCTTCTTGGGACTGATGCT 3′	475 bp
*IL6 rev*	5′ TGGAAATTGGGGTAGGAAGGAC 3′	
*TNF- α for*	5′ TAGCCCACGTCGTAGCAAAC 3′	566 bp
*TNF- α rev*	5′ ACCCTGAGCCATAATCCCCT 3′	
*PTGS2 for*	5′ TCACGTGGAGTCCGCTTTAC 3′	467 bp
*PTGS2 rev*	5′ AACTTCGCAGGAAGGGGATG 3‘	
*SLC7A11 for*	5′ GAAGCTGAGCTGGTGTGTAATG 3′	399 bp
*SLC7A11 rev*	5′ CTCCCGCTCATATTGCCCTG 3′	
*MMP2 for*	5′ CAGGGAATGAGTACTGGGTCTAT 3′	445 bp
*MMP2 rev*	5′ GGCTGCTTCACATCCTTCAC 3′	
*MMP9 for*	5′ TTCACCGGCTAAACCACCTC 3′	270 bp
*MMP9 rev*	5′ TAACGCCCAGTAGAGAGCCT 3′	
*Actin for*	5′ TATGCCAACACAGTGCTGTCTGG 3′	247 bp
*Actin rev*	5′ AGAAGCACTTGCGGTGCACGATG 3′	

### Cytokine Array

For the cytokine array media were prepared as previously described in section Conditioning of Hippocampus Medium. Then, the Proteome Profiler Mouse Cytokine Array Panel A (R&D Systems, Cat. No.: ARY006) was used based on the manufacturer's protocol. Here, 500 μl of the media were utilized per experimental repetition and per membrane. The chemiluminescence signals were measured using a chemiluminescence reader (Biostep) with an exposure time of 1 min. Quantification of signal intensity was performed using the ImageJ software. The quantified signal intensities were set in relation to internal positive controls of the cytokine array.

### Microscopy

The immunocytochemically stained microglia were recorded using a fluorescence microscope (AxioPlan 2, Zeiss). Additionally, an affiliated digital camera (AxioCam MRm, Zeiss) was used. For the data documentation the AxioVision 4.5 software (Zeiss) was utilized. Recordings of glutamatergic and GABAergic structural synapses were performed using a confocal laser scanning-microscope [LSM 510 Meta (Zeiss)]. Here, 5 stacks were adjusted for each recording with a width of 0.25 μm for each stack. Then, a maximum intensity projection was created by the overlay of 5 stack images. Values for gain, pinhole and threshold were kept constant for all recordings.

### Data Quantification

The analysis of microglial activation and apoptosis was performed with slides blinded for the observer and recorded at the fluorescence microscope. The remaining experiments were carried out without blinded groups. The percentual amount of activated microglia was determined using the Multi point tool in ImageJ (version 1.52 h). Here, completely rounded cells were declared as active/amoeboid and set in relation to the total number of microglia per image. Using this principle, the amount of Caspase-3 positive microglia, which were declared as apoptotic, was determined.

For the RT-PCR results, the rectangle tool in ImageJ was used. The mean gray value of every sample was measured and subtracted by the background. Finally, the values were set relative to the actin signal. The quantification of the cytokine array analysis was similarly performed. Here, the oval tool was used instead of the rectangle tool. The mean gray value of each sample was set in relation to the positive controls in the corner on the membrane after subtracting the background.

The quantification of synaptic puncta was performed using the “Puncta Analyzer” Plugin for ImageJ from Barry Wark (licensed under http://www.gnu.org/copyleft/gpl.html). Following settings were adjusted for the detection of puncta: rolling ball radius = 50 pixel, size (pixel^2^) = 2-infinity and circularity = 0.00–1.00. LSM recordings used for puncta analysis had a resolution of 2048 × 2048 pixel.

PNN area sizes were quantified using the “Freehand Selections” tool in ImageJ after defining a distance length depending on the scale bar. The fluorescence intensity of PNNs was analyzed by measuring the mean gray value and on the other hand by determining the total corrected total cell fluorescence (CTCF), as previously described (McCloy et al., 2014; Wegrzyn et al., 2020).

The recording and quantification parameters concerning the multielectrode array measurements can be found in Astrocyte-Neuron Co-Culture on MEAs in detail.

### Statistics

For the statistical *analysis of data sets GraphPad Prism (version* 8.2.1) was used. First, data sets were analyzed regarding their distribution using the Shapiro-Wilk or the Kolmogorov-Smirnov test. When data sets were normally distributed *p*-values were determined either with the *t*-test for two groups or via One-way ANOVA with additional Bonferroni multiple comparison test for more than two groups. If data sets were not normally distributed, the Mann-Whitney *U*-test or the Kruskal-Wallis test was used with a Dunn's multiple comparison test. The significance level was adjusted at *p* ≤ 0.05. The total number of experimental repetitions (biological replicates) varied between three and five depending on the experiment and can be found given as “N” in the corresponding figure legends. For immunocytochemical analysis two coverslips were used per experiment (technical replicates) and for MEA measurements one MEA-plate was used per experiment. In the result part data are given as mean ± SEM and in the figures as mean values with standard deviation (±SD), except the electrophysiological data which are presented as mean values with standard error of the mean (SEM). Additional information is given at the end of figure legends.

## Results

### Poly I:C Treatment Induces Microglial Activation and Release of Cytokines

**Figure 1:**
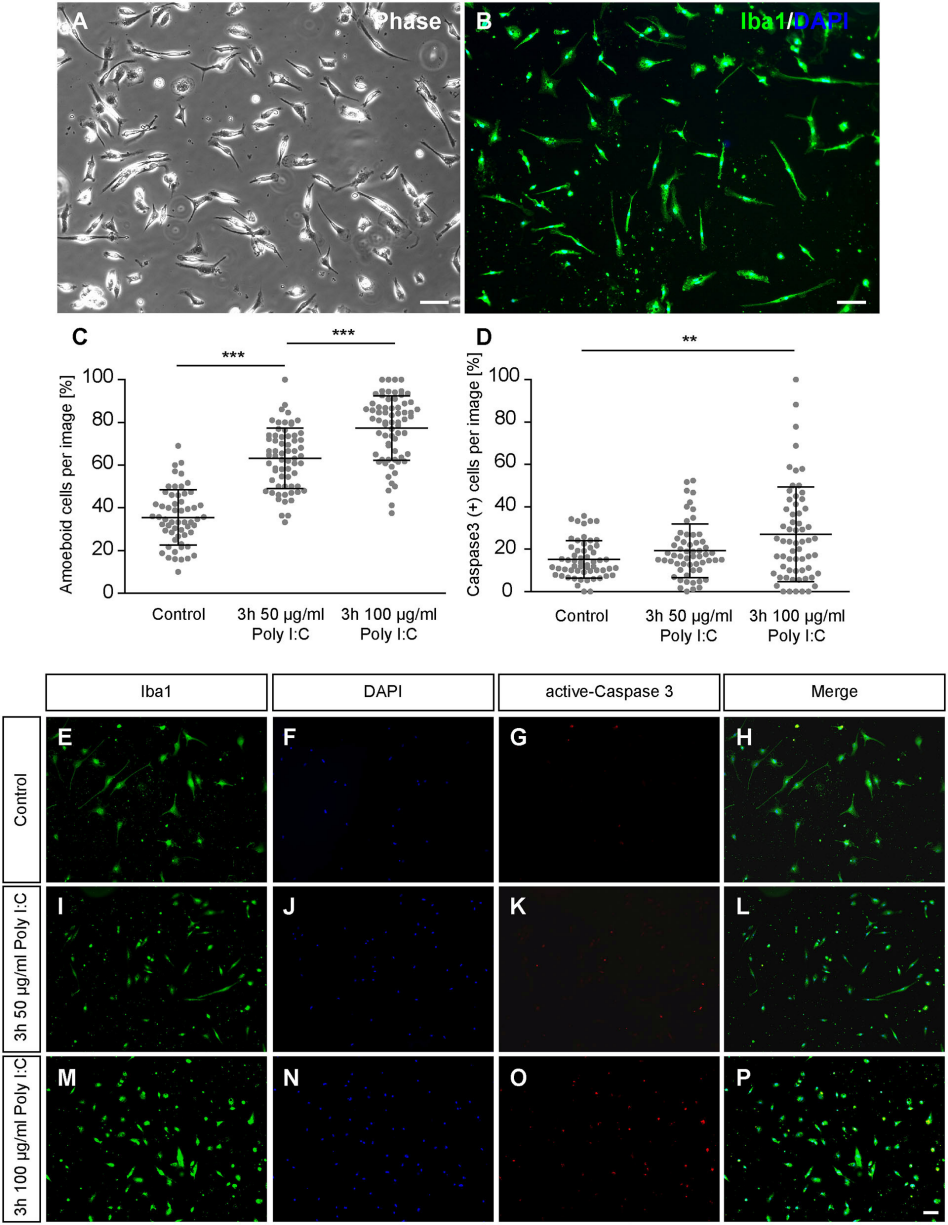
**(A,B)** Primary microglia were cultured for 2 days on PDL-coated coverslips and recorded via phase contrast microscopy. Additionally, microglia were immunostained against the typical microglia marker Iba1; **(C)** After 2 days in culture microglia were treated for 3 h with 50 or 100 μg/ml Poly I:C. Control conditions received an equal volume of microglia medium without Poly I:C. The amount of completely rounded microglia significantly raised with increasing concentrations of Poly I:C. Each dot is representing the percentage of completely rounded microglia determined per quantified recording; **(D)** Concurrently the percentage of apoptotic microglia was determined via immunocytochemical active Caspase 3 staining. A treatment with 100 μg/ml Poly I:C induced a significant increase of apoptotic microglia. Dots are representing the percentage of Caspase 3 positive microglia per analyzed image; **(E–P)** Representative fluorescence images of microglia double-stained against Iba1 and active Caspase 3 after Poly I:C treatment. An increase of rounded microglia as well as apoptotic microglia could be noticed when Poly I:C was added. Statistics: Three independent experiments (*N* = 3) were performed. In total 1505–1535 cells per condition were counted for microglia activation and 2005–2090 cells per condition were counted for apoptosis analysis. Data are shown as mean ± SD (One-way ANOVA with Bonferroni multiple comparison was used for microglial activation and Kruskal-Wallis test with Dunn's multiple comparison for the apoptosis analysis, *p ≤ 0.05, **p ≤ 0.01 and ***p ≤ 0.001). Scale bars: 100 μm.

After 2 days of culturing primary cortical microglia were treated for 3 h either with saline or Poly I:C with a concentration of 50 μg/ml or 100 μg/ml. Immunocytochemical staining revealed a positive signal for Iba1. In addition, cells showed a typical microglial morphology *in vitro* (Figures 1A,B), that has been already described in previous studies (Zhu et al., 2010; Lian et al., 2016; Rustenhoven et al., 2016; Zhao et al., 2018) and showed a phagocytic activity (Supplementary Video 1). Microglia of the control condition were mainly rod shaped, while microglia treated with increasing concentrations of Poly I:C displayed a rounded morphology with a visibly reduced cell surface (Figures 1E–P). Completely rounded Iba1 positive cells were defined as amoeboid and the percental fraction was determined in the cultures (Figure 1C). Cultures treated with saline contained 35.54 ± 1.70% completely rounded microglia. In contrast, a significantly increased amount of amoeboid microglia was observed when 50 μg/ml Poly I:C (*p* < 0.0001) were added to the culture medium. Here, cultures contained 63.20 ± 1.77% round shaped microglia. Microglia treated with 100 μg/ml Poly I:C developed a higher amount of amoeboid microglia (77.45 ± 1.84%; *p* < 0.0001).

To prevent unwanted effects caused by cytotoxic concentrations of Poly I:C, apoptosis rates were investigated via active-Caspase 3 staining (Figures 1E–P). In the control condition, 15.26 ± 1.18% apoptotic microglia were observed (Figure 1D). When microglia were treated for 3 h with 50 μg/ml Poly I:C, 19.25 ± 1.69% Caspase 3 positive microglia were registered in the cultures. The highest number of apoptotic cells was observed when microglia were incubated with 100 μg/ml Poly I:C. Here, 26.99 ± 2.92% of the microglia were apoptotic and significantly increased in comparison to the control condition (*p* = 0.0058). Because of the increasing apoptosis rate, following experiments were performed with a Poly I:C concentration of 50 μg/ml.

To determine if microglia cultured for 2 days and subsequently incubated for 24 h with 50 μg/ml Poly I:C expressed inflammation related genes, RT-PCR was performed in this preparation. After lysis, cDNA synthesis and RT-PCR analysis were performed. In the microglial cultures, we observed the expression of Interleukin 1β (*IL-1*β, 0.98 ± 0.01), Interleukin 6 (*IL-6*, 0.52 ± 0.05), and tumor necrosis factor α (*TNF-*α, 0.82 ± 0.09). In addition, the extracellular matrix modulating members of the matrix metalloproteinase family *MMP2* (0.11 ± 0.05) and *MMP9* (0.25 ± 0.05) were also detected. *Prostaglandin-endoperoxide synthase 2* (*PTGS-2*) also known as Cyclooxygenase 2 could be identified with a low expression level of 0.10 ± 0.04 relative to *actin*. Last, *soluble carrier family 7 member 11* (*SLC7A11*) which is a member of a heteromeric transport system of glutamate and glycine in the membrane of microglia was detected with a relative expression of 0.66 ± 0.08.

**Figure 2:**
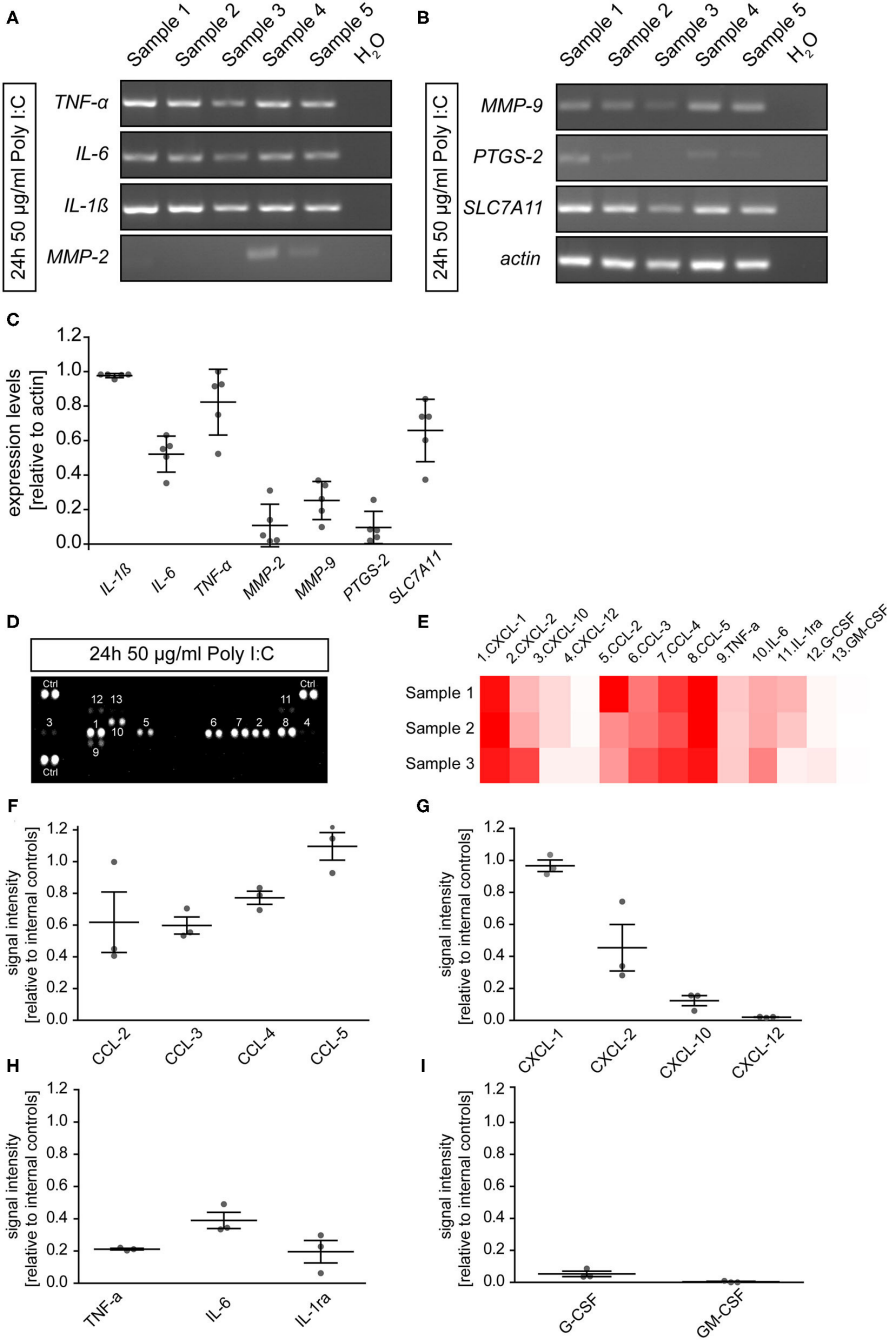
**(A,B)** Microglia were cultured for 2 days and subsequently treated with 50 μg/ml Poly I:C for 24 h. Afterwards, cells were lysed, and RT-PCR analysis was performed. Microglia expressed cytokines like *IL-1*β, *IL-6*, and *TNF-*α. In addition, members of the matrix metalloproteinase family *MMP2* and *MMP9* were expressed. The antiporter subunit *SLC7A11* which convey glutamate release from activated microglia was present on mRNA level after Poly I:C stimulation. Last, expression of *PTGS2* which is encoding for cyclooxygenase 2 could be observed; **(C)** The band intensities were quantified and set in relation to *actin*; **(D,E)** Cytokine array analysis was utilized to identify microglial secreted factors in the supernatant after a 24 h activation with 50 μg/ml Poly I:C. Dots are representing signals of cytokine detecting antibody pairs on the array membrane. More intense signals are indicating higher protein levels in the supernatant. The corresponding cytokine to the number above or under a dot pair can be found in the heatmap (red = high levels; white = low levels) **(D)**. Members of the CXCL and CCL chemokine families as well as IL-6 and TNF-α could be observed in the supernatant of activated microglia. Especially CCL5 and CXCL1 were strongly accumulated in the supernatant; **(F–I)** Signal intensities were quantified and presented relatively to the internal cytokine array positive controls. Statistics: For the RT-PCR analysis five experimental repetitions (*N* = 5) were performed and for the cytokine array analysis the supernatants of three independently prepared microglia cultures (*N* = 3) were used.

To verify the presence of immune factors on the protein level, a proteome profiler was utilized. In total, 13 of 40 analyzed secreted cytokines could be detected in the supernatant of microglial cells after an incubation time of 24 h with 50 μg/ml Poly I:C (Figures 2D–I). The pattern of cytokines in the supernatant was similar to a previously published study containing a cytokine array of primary microglia activated for 24 h with soluble Tenascin-C or LPS (Haage et al., 2019). The strongest signals could be observed for the CC-motif-chemokine ligands (CCL). Here, CCL-5 showed the most intense signal (1.10 ± 0.09) in relation to internal positive controls of the array, followed by CCL-4 (0.77 ± 0.04), CCL-3 (0.60 ± 0.05), and CCL-2 (0.62 ± 0.19). In addition, chemokine (C-X-C-motif) ligand 1 (CXCL-1, 0.97 ± 0.04) and 2 (0.45 ± 0.14), were strongly present in the culture medium. As previously identified on mRNA level, TNF-α (0.21 ± 0.01) and IL-6 (0.39 ± 0.05) were also detected in the supernatant. The remaining factors were only present in relatively low concentrations (CXCL-10: 0.12 ± 0.03; CXCL-12: 0.02 ± 0.001; G-CSF: 0.05 ± 0.02; GM-CSF: 0.003 ± 0.003; IL1-ra: 0.20 ± 0.07). Interestingly, the strongly detected signal for IL-1β in the RT-PCR could not be observed in the supernatant on the protein level.

### Microglial Secreted Factors Increase Spontaneous Network Activity of Cultured Neurons

**Figure 3:**
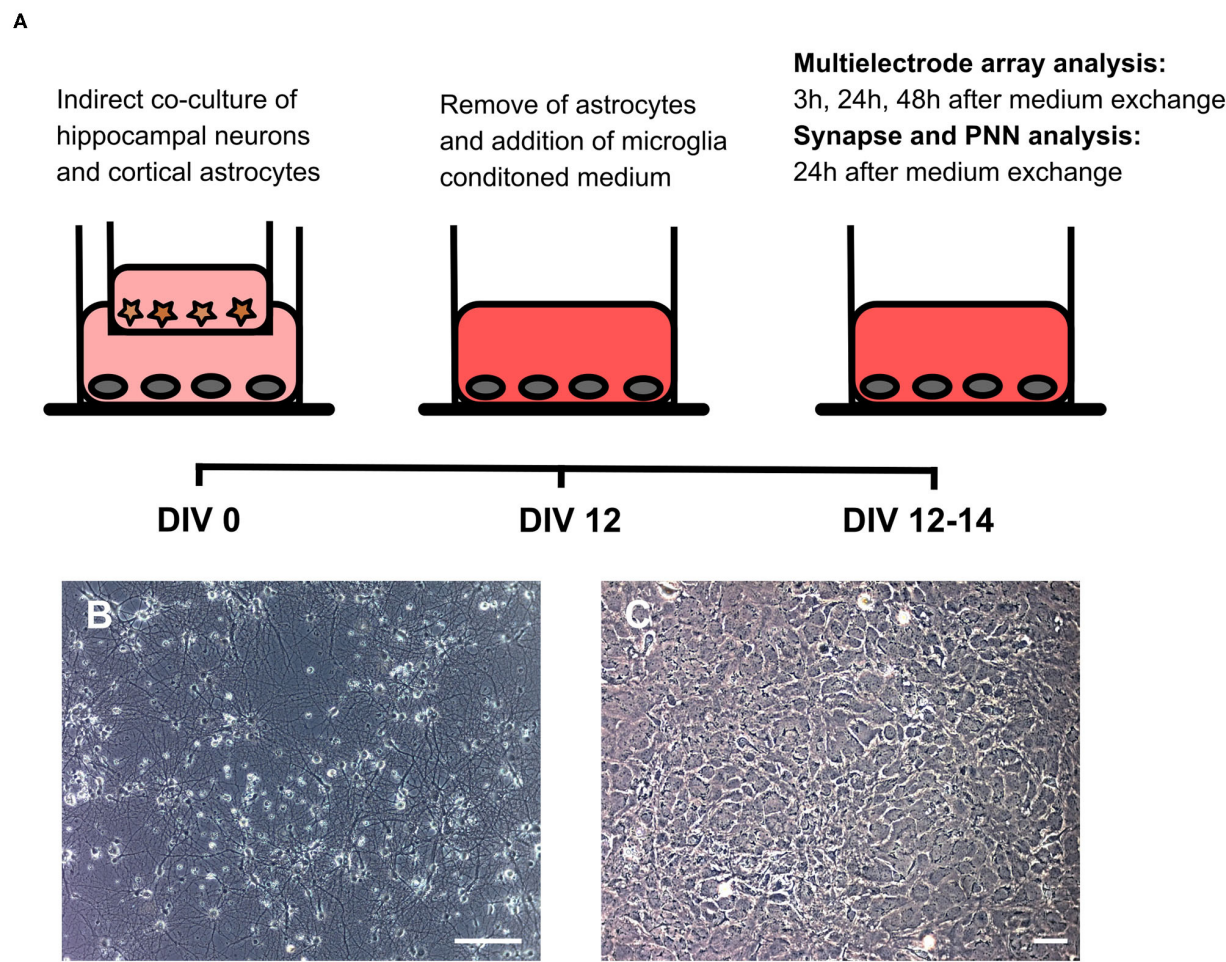
**(A)** Native hippocampal neurons were cultured on MEAs or well plates in an indirect co-culture setup with cortical astrocytes, previously cultured in transwells. This setup allowed for the culturing of pure neuronal cultures in serum-free medium. Since astrocytes express toll-like receptors on their surface, transwells were removed after 12 days *in vitro* before treatment with microglial conditioned medium. Baseline MEA recordings were performed and subsequently 25% (v/v) of the culture medium was replaced by microglia conditioned medium or by hippocampus medium as control. Next MEA recordings were performed 3, 24, and 48 h after the medium exchange. Neurons cultured in well plates were fixed after 24 h for structural synapse and PNN analysis since MEA recordings revealed the strongest effect at this point in time; **(B)** Representative phase contrast image of a hippocampal neuronal culture after 14 days *in vitro* (scale bar: 100 μm); **(C)** Representative phase contrast image of cortical astrocytes forming a monolayer (scale bar: 100 μm).

**Figure 4:**
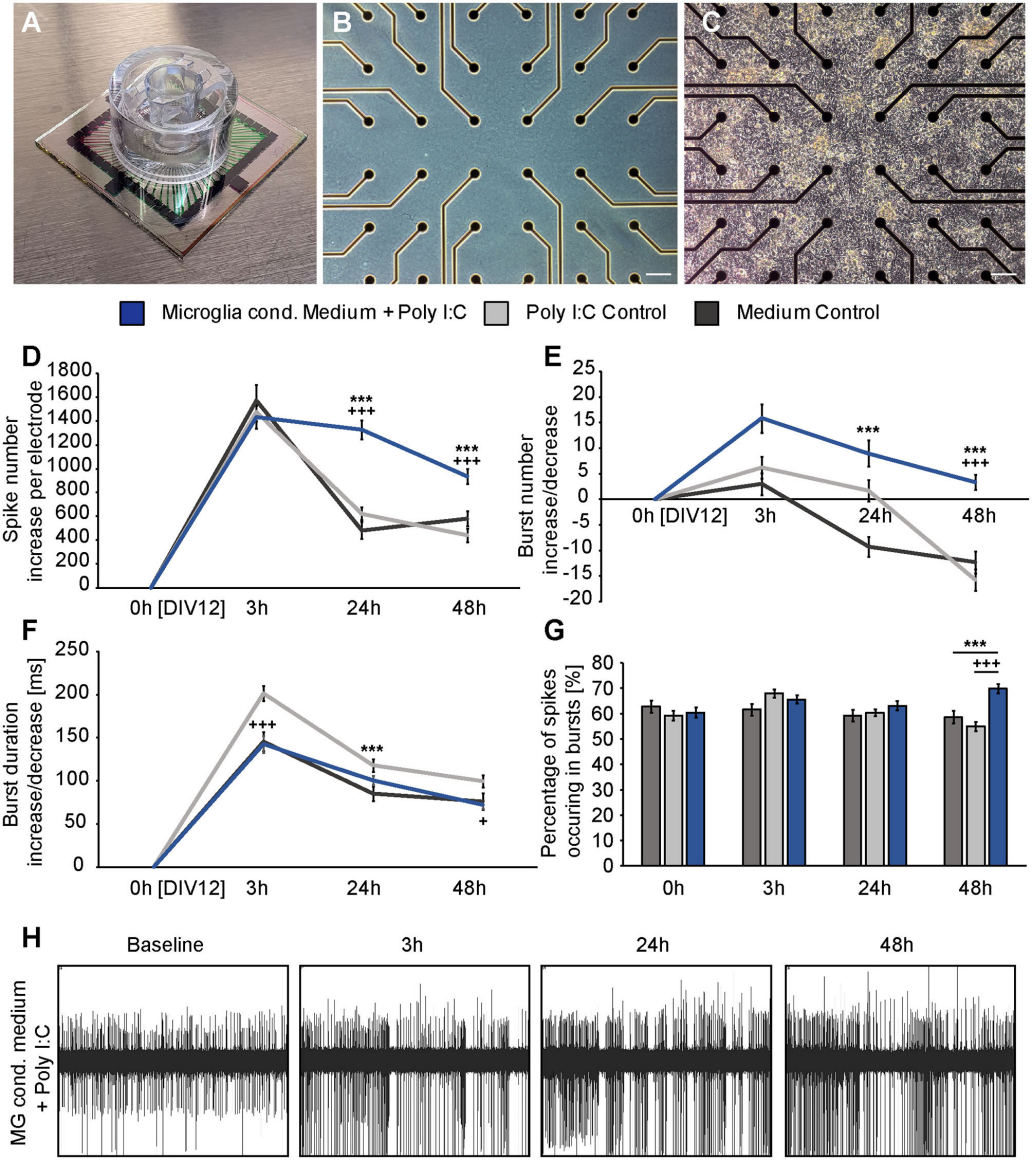
**(A)** Standard 60MEA200/30iR-Ti-gr with a custom manufactured transwell holder on top; **(B)** Phase contrast image of an empty electrode field without neurons; **(C)** Phase contrast image of a dense neuronal network cultured on the electrode field of a MEA; **(D)** The absolute increase of single occurring action potentials, so-called spikes was analyzed 3, 24, and 48 h after administration of previously conditioned medium of Poly I:C activated microglia (blue), sole fresh medium (dark gray) or Poly I:C containing medium (light gray). A significant increase in the number of spikes could be detected after 24 h and 48 h when neurons were treated with microglia conditioned medium (* represents significant to medium control, + represents significant to Poly I:C control). In contrast, both control conditions nearly reached the baseline level after 24 and 48 h; **(E)** The increase of organized network firings, so-called bursts, after the medium exchange was determined. As previously, the strongest increase was observed in the condition with microglia conditioned medium; **(F)** Burst duration analysis did not reveal significant changes between neuronal cultures treated with microglia conditioned medium and culture medium. However, Poly I:C containing medium increased the burst duration significantly after 3 h compared to neurons treated with microglia conditioned medium and after 24 h compared to the medium control; **(G)** The percentage of spikes in bursts was just increased after 48 h in the microglia conditioned medium group; **(H)** Representative MEA recording of one electrode after treatment with microglia conditioned medium. The increasing number of recorded spikes can clearly be seen. Statistics: Five independent experiments (*N* = 5) were performed for the medium control and Poly I:C control. Four independently prepared cultures (*N* = 4) were treated with microglia conditioned medium. In total, data of 240–300 electrodes were considered for quantification. Data are shown as mean ± SEM (Kruskal-Wallis test with Dunn's multiple comparison, *p ≤ 0.05, **p ≤ 0.01 and ***p ≤ 0.001; ^+^*p* ≤ 0.05, ^++^*p* ≤ 0.01 and ^+++^*p* ≤ 0.001). Here, the Kruskal-Wallis test was separately performed for each recording time point. A comparison of the mean values between time points was not included. Scale bar: 100 μm.

To analyze the impact of microglia secreted factors on a whole cultured neuronal network, hippocampal neurons were kept for 12 days in an indirect contact with cortical astrocytes. In this setup, astrocytes served as a feeder layer and enabled the culturing of neurons in serum free medium on multielectrode arrays (Geissler and Faissner, 2012) (Figures 4A–C). After 12 days *in vitro* baseline recordings were performed. Then, inserts with astrocytes were removed and the medium was partially (25% v/v) changed. Neurons on multielectrode arrays were treated either with 24 h conditioned medium of microglia activated with Poly I:C, a medium control and a second medium control containing Poly I:C. Measurements were performed after 3, 24, and 48 h. A scheme of the experimental setup can be found in Figure 3.

Three hours after the medium exchange MEA recordings were performed and the absolute increase or decrease for every single electrode was calculated using the baseline measurement. In this way, the average absolute increase/decrease per electrode was compared between all groups (Figures 4D–F). First, the number of single occurring action potentials, so-called spikes, was analyzed. Neuronal networks of all conditions showed a raise in their spontaneous network activity 3 h after the medium exchange. The conditioned medium of stimulated microglia led to an absolute increase of 1433.73 ± 94.94 spikes per electrode. In comparison, neurons treated with medium showed an increase of 1573.54 ± 131.65 spikes per electrode and neurons treated with medium containing Poly I:C 1478.77 ± 79.58 spikes per electrode (Figure 4D). No significant differences could be observed between all groups. After 24 h neuronal networks of the medium control group reduced their spontaneous activity and showed 482.35 ± 69.30 more spikes than in the baseline recording. In the second control condition, that included Poly I:C an absolute increase of 621.88 ± 52.93 compared to the baseline measurement could be observed. Statistical analysis did not reveal a significant difference between both groups after 24 h (*p* = 0.639). However, neurons treated with the conditioned medium of stimulated microglia still showed a sustained increase in their network activity after 24 h and 1328.33 ± 80.70 more action potentials per electrode than in the baseline recording could be observed. Here, a statistical significance was detected in comparison to the medium control (*p* < 0.0001) and to the medium control with Poly I:C (*p* < 0.0001). After 48 h an increase of 581.89 ± 62.59 spikes in the medium control and of 441.82 ± 57.96 in the Poly I:C containing medium control was observed. As already described after 24 h, no significant change was present between both controls (*p* = 0.103) after 48 h. A significant higher number of spikes per electrode was observed for neurons treated with microglia conditioned medium after 48 h. Here, neuronal networks showed an average increase of 934.86 ± 62.43 (*p* = 0.0002 vs. medium control; *p* < 0.0001 vs. medium control with Poly I:C).

Next, the number of bursts was analyzed. Bursts are defined as a sequence of synchronously appearing action potentials and are indicating an organized communication of the network (Figure 4E). After 3 h all cultures showed an increase in their number of bursts compared to the baseline recording. However, the differences were not as high as previously observed for spikes. Neurons of the medium control showed an increase of 3.03 ± 2.13 bursts, while neurons treated with Poly I:C containing medium displayed an increase of 6.25 ± 2.16 bursts. The highest difference could be observed for neuronal networks treated with conditioned medium of stimulated microglia. Here, 15.90 ± 2.80 more bursts per electrode were registered than in the baseline recording. However, this increase was not significant in comparison to the medium control group (*p* = 0.077) and the Poly I:C containing medium control (*p* > 0.999). Between both control groups no significant changes could be observed (*p* = 0.057). After an incubation time of 24 h neuronal networks treated solely with medium formed – 9.23 ± 1.98 bursts than previously recorded in the baseline measurement. In comparison, networks incubated with Poly I:C containing medium showed 1.67 ± 2.12 more bursts and reached therefore nearly the same level as observed during baseline recordings. At this point in time, statistical analysis revealed a valid difference between both control groups (*p* = 0.045). The highest increase in burst number could be observed in neuronal networks treated with conditioned medium of stimulated microglia (8.98 ± 2.54). Here, a statistically significant increase could be observed when it was compared to the medium control (*p* < 0.0001) but not to the Poly I:C containing control (*p* = 0.101). After 48 h neuronal networks incubated with conditioned medium of Poly I:C treated microglia came closer to the activity level of the baseline recording and showed only 3.36 ± 1.47 more bursts per electrode. This increase was significantly higher compared to the medium control (−12.30 ± 2.13, *p* < 0.0001) and Poly I:C control (−15.72 ± 2.08, *p* < 0.0001). No significant differences could be observed between both control groups (*p* = 0.247).

The burst duration was analyzed after microglia conditioned medium was added (Figure 4F). As previously described for the burst number, the burst duration was not strongly affected by the medium exchange. Three hours after adding the different media, all conditions showed an increase in burst duration. Neurons treated simply with medium developed bursts that lasted 145.60 ± 11.08 ms longer than bursts in the baseline recording. Nearly the same value could be observed for neurons treated with microglia conditioned medium (142.48 ± 9.70 ms). Interestingly, Poly I:C containing medium increased the burst duration to a higher extent (201.40 ± 8.42 ms). This raise was significant compared to the medium control (*p* < 0.0001) and microglia conditioned medium (*p* < 0.0001). After 24 h similar mean values could be observed between all groups (medium control: 85.23 ± 8.42 ms; Poly I:C control:117.91 ± 7.38 ms; microglia conditioned medium: 100.64 ± 5.34). Here, the Poly I:C conditioned medium significantly increased the burst duration in comparison to the medium control (*p* < 0.0001). After 48 h neuronal networks treated with medium had 75.82 ± 9.52 ms longer bursts than recorded in the baseline measurement. Neurons treated with microglia conditioned medium showed a similar increase of 72.10 ± 4.92 ms in their burst duration without any significance (*p* > 0.9999). The Poly I:C containing medium increased the burst duration of neuronal networks also after 48 h. Here, an increase of 99.43 ± 7.04 ms per electrode in comparison to the baseline measurement was observed. This value was not significant compared to the medium control (75.82 ± 9.52 ms, *p* = 0.580) but slightly significant compared to the group that received microglia conditioned medium (72.10 ± 4.92 ms, *p* = 0.038).

Furthermore, we analyzed the percentage of spikes occurring in bursts (Figure 4G). The baseline recordings revealed a similar percentage of spikes occurring in bursts in all conditions (medium control: 62.90 ± 2.37%; Poly I:C control: 59.26 ± 1.89%; microglia conditioned medium: 60.50 ± 2.08%). After an incubation time of 3 h neurons treated with medium did not show a reaction regarding the percentage of spikes in bursts. Here, 61.54 ± 2.20% of all spikes occurred in bursts. Neurons incubated with Poly I:C containing medium or microglia conditioned medium showed a small raise in this parameter (Poly I:C control: 67.97 ± 1.65%; microglia conditioned medium: 65.62 ± 1.68%). After 24 h all cultures exhibited again similar mean values (medium control: 59.25 ± 2.23%; Poly I:C control: 60.41 ± 1.38%; microglia conditioned medium: 63.09 ± 1.85%). 48 h after the medium exchange neurons treated with microglia conditioned medium showed a remarkable increase in the percentage of spikes occurring in bursts (69.94 ± 1.76%). In contrast, neurons of both control conditions showed slightly reduced values in comparison to the baseline recording (medium control: 58.63 ± 2.42%; Poly I:C control: 54.97 ± 1.82%).

**Figure 5:**
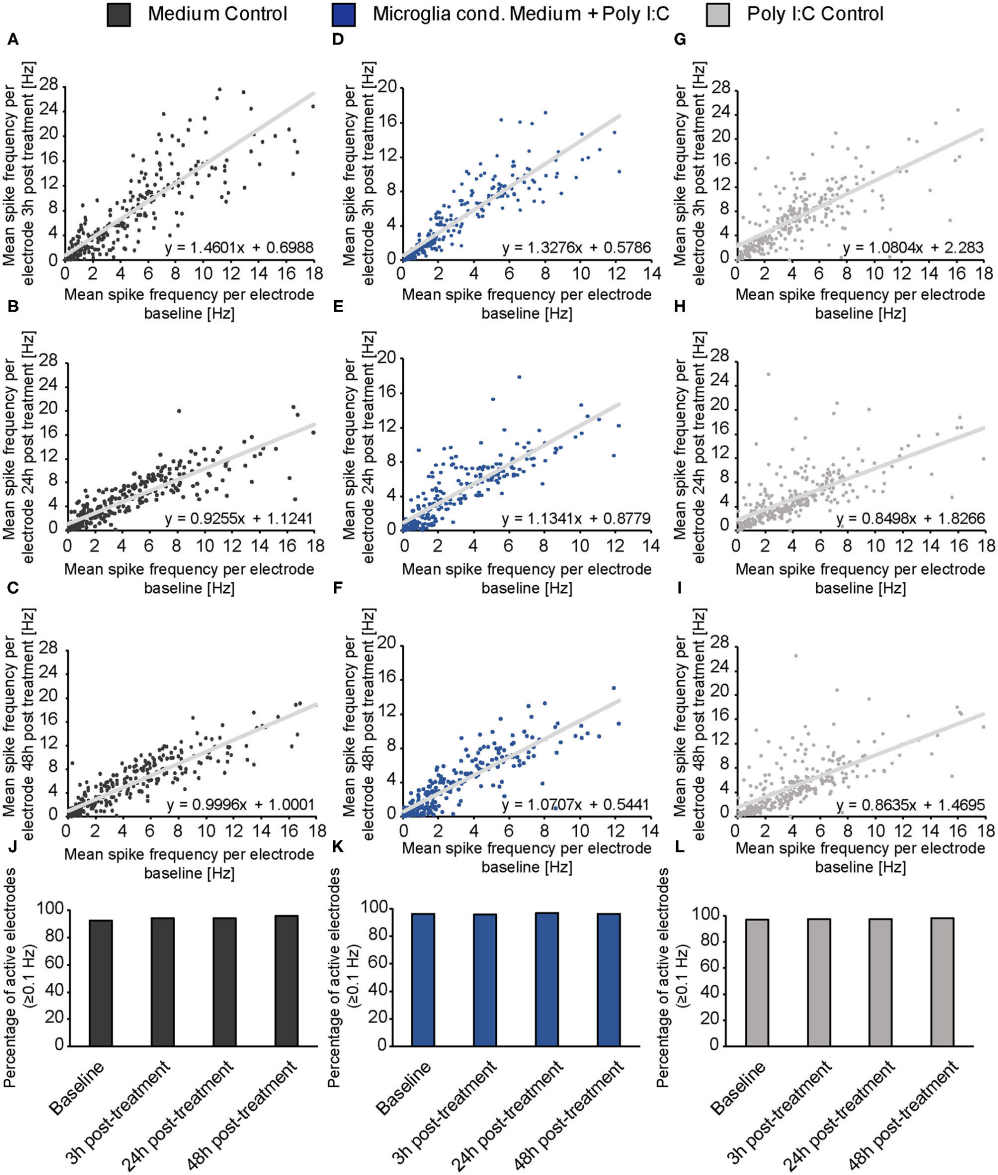
**(A–I)** The mean spike frequency (Hz) determined by every single electrode during the baseline measurement (x-axis) was plotted against the mean spike frequency recorded by the same electrode 3, 24, and 48 h after the treatment with neuron medium (dark gray), conditioned medium of activated microglia (blue) and Poly I:C containing medium (light gray) (y-axis). This data representation considers all electrodes and indicates an even increase in the activity of the neuronal population when microglia conditioned medium was administered. A noticeable increase of a neuronal subpopulation with specific firing characteristics (e.g., increase/decrease of merely fast spiking neurons) was not observed; **(J–L)** The percentage of active electrodes (≥0.1 Hz) was controlled during the experimental procedure for all conditions to exclude major failures of electrodes induced by apoptosis. The percentage of spiking electrodes remained constant during the whole experiment and reached between 92 and 98% active electrodes. Statistics: Five independent experiments (*N* = 5) were performed for the medium control and Poly I:C control. Four independently prepared cultures (*N* = 4) were treated with microglia conditioned medium. In total, data of 240–300 electrodes are presented in the plots.

To clarify if the increase of the spontaneous network activity of neurons treated with microglia conditioned medium is caused by an overall raise of activity and therefore a reaction of the whole network or if it is caused by an activity increase of neuronal subpopulations (e.g., fast spiking interneurons), mean baseline spike frequency of every single electrode was plotted against the mean spike frequency of same electrode after 3, 24, and 48 h of treatment (Figures 5A–I). For neurons treated with conditioned medium of activated microglia, an even increase of the spike frequency was observed after 3, 24, and 48 h of treatment (Figures 5D–F). The increase did not depend on the spike frequency of the baseline measurement indicating an overall reaction of the neuronal network on treatment. A specific subpopulation of neurons which separately increased their firing was not observed. The medium control condition showed also an even increase in spike frequency after 3 h (Figure 5A). However, after 24 and 48 h mean frequency of single electrodes returned to lower levels as previously observed in Figure 4 (Figures 5B,C). With regard to the Poly I:C control group, most electrodes showed a similar reaction after 3 h compared to the medium control and microglia conditioned medium group (Figure 5G). However, separate electrodes still showed an increased firing after 24 and 48 h while most electrodes lowered their activity, especially those with a baseline frequency of 2–6 Hz (Figures 5H,I).

**Figure 6:**
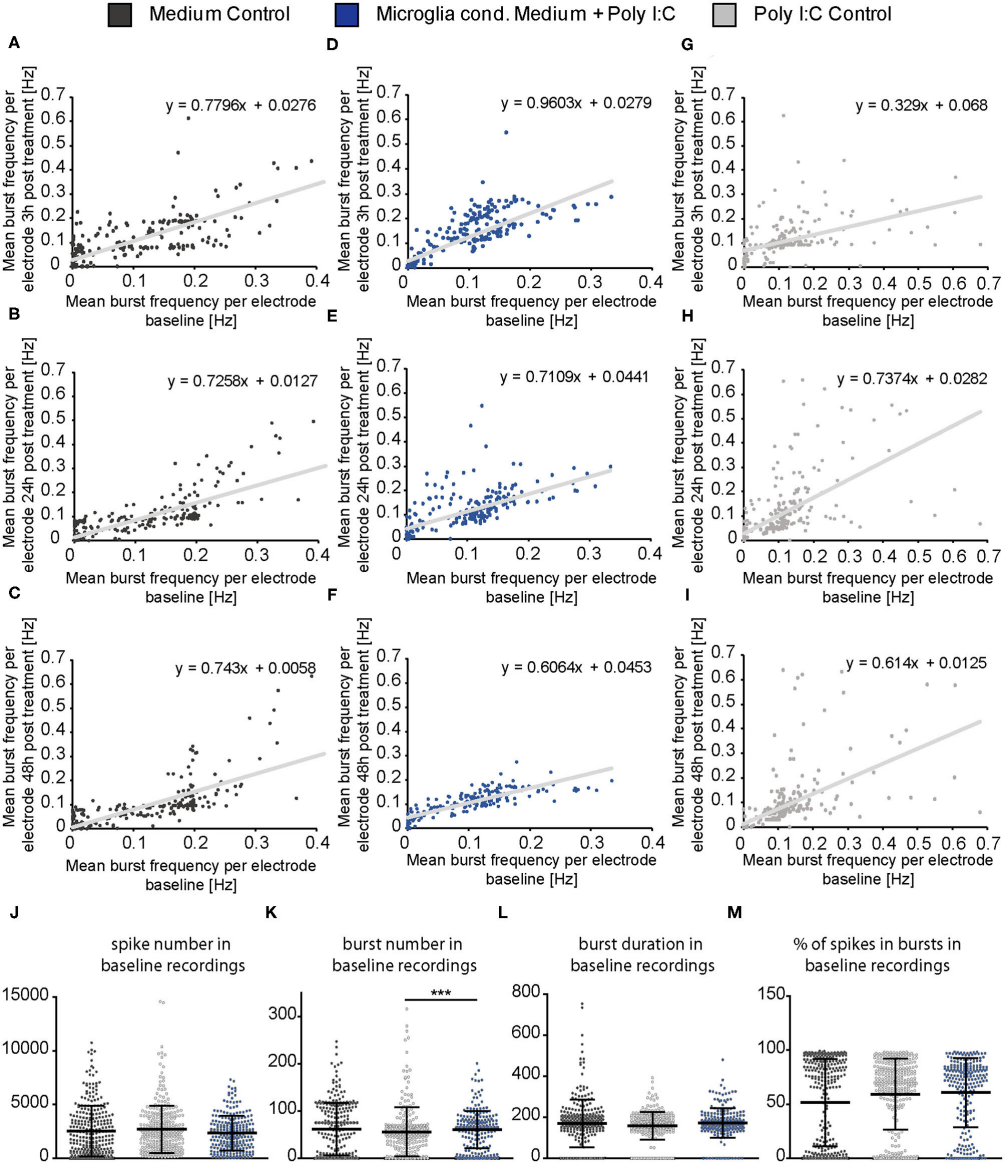
**(A–I)** The mean burst frequency (Hz) measured by every MEA electrode during the baseline recording (x-axis) was plotted against the mean burst frequency determined by the same electrode after a 3, 24, and 48 h treatment with neuron medium (dark gray), conditioned medium of activated microglia (blue) and Poly I:C containing medium (light gray)(y-axis). As previously described in figure legend 5 an even increase of the burst activity in the neuronal population could be observed, independently of a low, middle, or high firing frequency in the baseline measurement; **(J–M)** Detailed representation of baseline measurements. To ensure the comparability of the MEA data, baseline measurements of each experimental condition were compared to each other. The mean values of the spike number, the burst duration (ms) and the percentage of spikes in bursts were similar to each other in the baseline recordings and did not show significant changes between all three groups. The mean value of the burst number was slightly higher in the baseline of the microglia conditioned medium group in comparison to the Poly I:C control group (*p* < 0.0001). Further significant changes were not observed with regard to the average burst number in the baseline recordings. Statistics: Five independent experiments (*N* = 5) were performed for the medium control and Poly I:C control. Four independently prepared cultures (*N* = 4) were treated with microglia conditioned medium. In total, data of 240–300 electrodes were considered for baseline analysis. Data in **(J–M)** are shown as mean ± SD (Kruskal-Wallis test with Dunn's multiple comparison, *p ≤ 0.05, **p ≤ 0.01 and ***p ≤ 0.001).

Same representation of data was performed for the mean burst frequency (Figures 6A–I). After 3 h of treatment, the medium control group and the Poly I:C control group showed only a minor increase of burst frequency measured by single electrodes (Figures 6A,G). However, neurons treated with microglia conditioned medium showed an increase in burst frequency of neurons with a previously measured baseline burst frequency of 0.05–0.2 Hz (Figure 6D). This increase was also visible after 24 h (Figure 6E) but vanished after 48 h (Figure 6F) similar to the observation made in Figure 4E. A subpopulation of high frequently firing neurons which specifically responded to the treatment was not detected. Here, also an even increase of burst activity was observed. As previously described for the spike frequency of the Poly I:C control group, single electrodes recorded a high frequently burst firing after 24 and 48 h while most other electrodes returned to a lower level (Figures 6H,I). This was not observed in the medium control group (Figures 6B,C).

In order to control if apoptotic events and the consequent loss of larger neuronal network regions influenced the electrophysiological data, the percentage of active electrodes (spike frequency ≥0.1 Hz) was determined for all conditions (Figures 5J–L). Here, the percentage of active electrodes remained constant in all three conditions over the entire experimental procedure and reached values between 92 and 98% active electrodes. For reasons of comparability, baseline characteristics of all groups and all analyzed parameters were compared to each other (Figures 6J–M). In the baseline recordings following mean values could be observed for the spike numbers: 2508.99 ± 135.80 spikes for the medium control group, 2629.44 ± 126.81 spikes for the Poly I:C control group, 2307.74 ± 103.75 spikes for the microglia conditioned medium group. No significant differences were observed between all three groups. The following mean values for number of bursts were detected in the baseline recording: 61.52 ± 3.10 bursts for the medium control group, 55.56 ± 3.01 bursts for the Poly I:C control group, 60.46 ± 2.56 bursts for the microglia conditioned medium group. Here, a significant difference could be observed between the Poly I:C control group and the microglia conditioned medium group (*p* < 0.0001). Next, burst duration was compared between the conditions and revealed following mean values: 168.24 ± 7.21 ms in the medium control group, 158.27 ± 3.90 ms in the Poly I:C control group, and 167.90 ± 6.13 ms in the microglia conditioned medium group. No significant changes were observed for this parameter. Last, percentage of spikes in bursts was compared in the baseline recordings. Here, the following mean values were observed: 62.90 ± 2.36% for the medium control group, 59.26 ± 1.90% for Poly I:C control group, and 60.50 ± 2.08% for microglia conditioned medium group. Here, also no significant alterations were detectable between all groups.

### Treatment With Microglial Conditioned Medium Impairs Perineuronal Net Structure and Alters Synaptic Balance

Since we observed a distinct increase in neuronal activity after the administration of microglia conditioned medium, we decided to investigate possible effects on structural synapse numbers and PNNs. Therefore, hippocampal neurons were cultured for 12 days and a 25% (v/v) medium exchange was performed, as previously described for the MEA experiments. Neurons were fixed after 24 h since we observed the strongest effects in our MEA recordings at this point in time. Then, immunocytochemical staining against different synaptic markers and Aggrecan, a main component of PNNs, was performed for the analysis of structural synapse numbers on PNN positive and negative neurons. Here, we focused our synapse analysis on the neuronal soma region including proximal dendrites. Neurons defined as PNN negative were recorded from cultures also stained against Aggrecan and were therefore excluded to be putative PNN positive.

**Figure 7:**
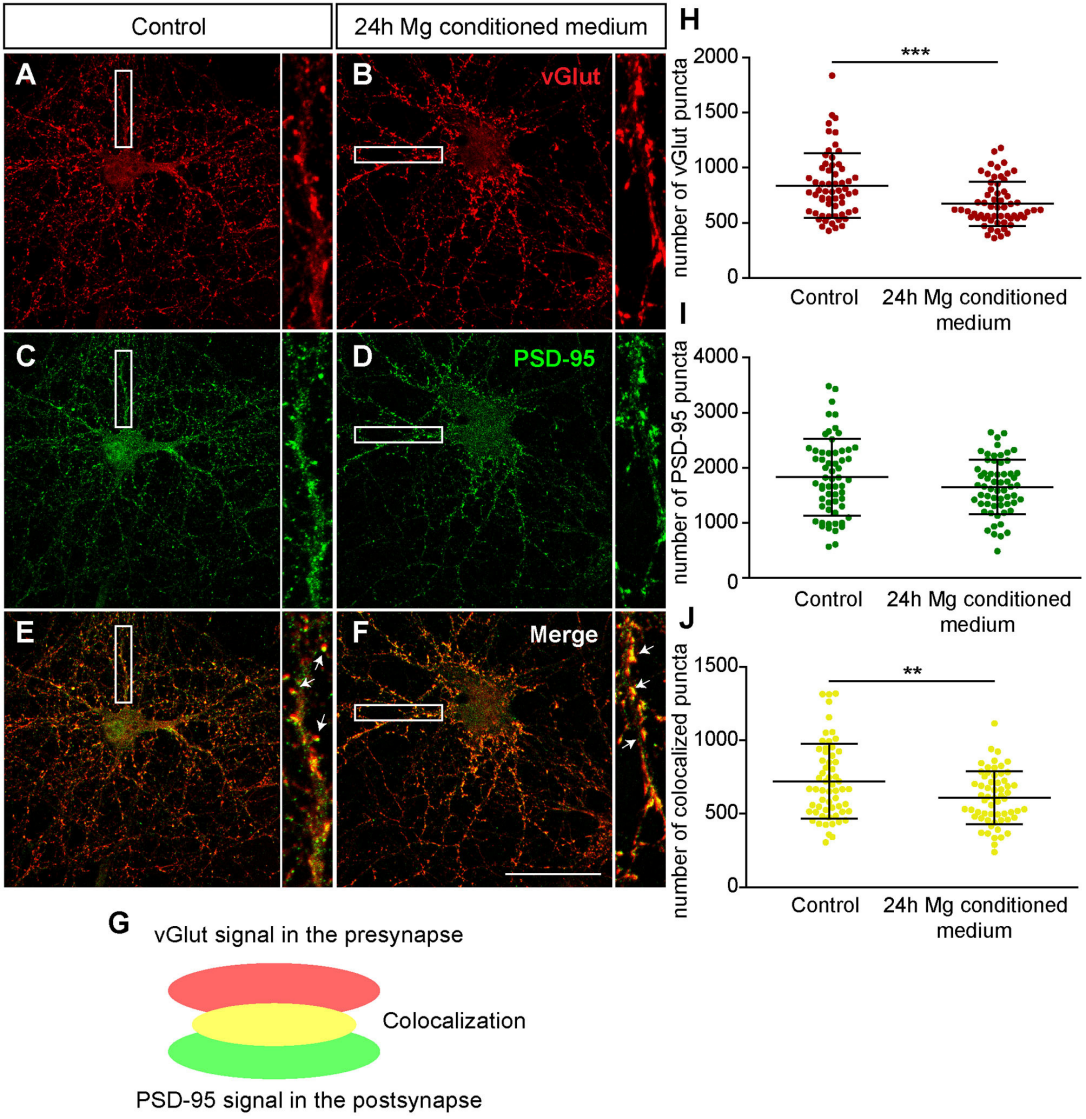
**(A–F)** Confocal laser-scanning microscopy of immunocytochemically visualized presynaptic vGlut (red) and postsynaptic PSD-95 puncta (green) on hippocampal neurons treated for 24 h with conditioned medium of Poly I:C activated microglia or hippocampus medium as control. Before treatment, neuronal networks were cultured for 12 days; **(E–G)** A colocalization of presynaptic vGlut and postsynaptic PSD-95 puncta yielded in yellow signals (indicated by arrows) which were defined as structural glutamatergic synapses; **(H)** Quantification of vGlut puncta revealed a significant reduction when neurons were treated with microglia conditioned medium; **(I)** With regard to PSD-95 puncta no significant changes were observed after treatment; **(J)** The number of colocalized synaptic puncta was significantly lower in neuronal cultures treated with microglia conditioned medium (scale bar: 50 μm). Statistics: Three independent experimental repetitions (*N* = 3) were performed for the analysis of synaptic puncta. For the quantification 20 neurons (*n* = 20) were recorded per repetition. Data are shown as mean ± SD (t-test, *p ≤ 0.05, **p ≤ 0.01 and ***p ≤ 0.001). Single dots in diagram **(H–J)** are representing puncta numbers of individually analyzed neurons.

**Figure 8:**
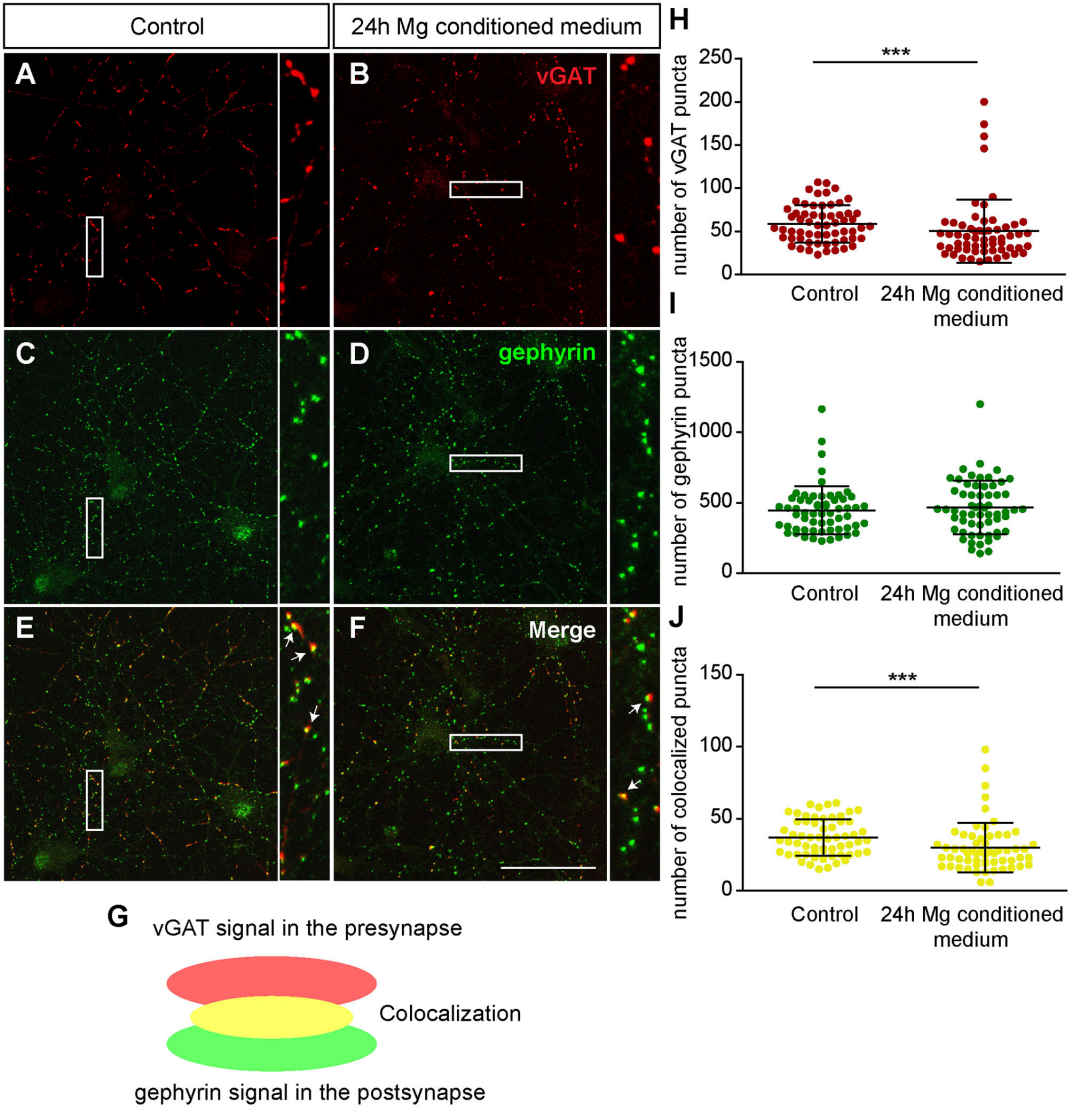
**(A–F)** Confocal laser-scanning microscopy of immunocytochemically visualized presynaptic vGAT (red) and postsynaptic gephyrin puncta (green) on cultured hippocampal neurons incubated for 24 h with microglia conditioned medium or hippocampus medium. Neurons were previously kept for 12 days *in vitro*; **(E–G)** A colocalization of vGAT and gephyrin puncta led to yellow signals in maximum intensity projection (indicated by arrows) which were defined as structural GABAergic synapses; **(H)** Analysis of presynaptic vGAT puncta revealed a significant reduction in neuronal cultures treated for 24 h with microglia conditioned medium in comparison to neuronal cultures treated with hippocampus medium; **(I)** No significant changes were observed with regard to the number of postsynaptic gephyrin puncta between both conditions; **(J)** The number of colocalized inhibitory puncta was significantly lower when neurons were incubated with conditioned medium of Poly I:C activated microglia (scale bar: 50 μm). Statistics: Three independent experimental repetitions (*N* = 3) were performed for the analysis of synaptic puncta. For the quantification 20 neurons (*n* = 20) were recorded per repetition. Data are shown as mean ± SD (Mann-Whitney *U*-test, *p ≤ 0.05, **p ≤ 0.01 and ***p ≤ 0.001). Dots in diagram **(H–J)** stand for puncta numbers of single neurons, as previously described in figure legend 5.

First, we focused on neurons which were negative for Aggrecan and consequently declared as PNN-negative. When neurons were treated for 24 h with microglia conditioned medium a significantly reduced number of presynaptic vGlut puncta (672.72 ± 25.85; *p* < 0.0001) was determined in comparison to neurons treated solely with hippocampus medium (836.97 ± 37.82)(Figures 7A,B,H). In contrast no significant change was observed for the number of postsynaptic PSD-95 puncta. Here, neurons of the control condition formed 1830.07 ± 89.57 and neurons of the treated conditioned 1652.72 ± 63.45 puncta (*p* = 0.1088) (Figures 7C,D,I). Colocalizations of presynaptic and postsynaptic puncta yielded in yellow signals that were defined as a structural glutamatergic synapses (Figures 7E–G). While neurons of the control conditioned formed 720.85 ± 32.99 colocalized puncta, neurons treated with microglia conditioned medium showed a significant reduction with only 607.95 ± 23.23 colocalized signals (*p* = 0.0060) (Figure 7J). In addition, inhibitory synapse numbers were analyzed via confocal laser-scanning microscopy (Figures 8A–F). For the presynaptic marker vGAT, 58.93 ± 2.80 puncta were observed when neurons were merely incubated with fresh medium. In contrast, neurons treated with microglia conditioned medium showed a mild but significant reduction in vGAT puncta (50.35 ± 4.72, *p* = 0.0006) (Figures 8A,B,H). In contrast, no significant change could be observed for postsynaptic gephyrin puncta (Control: 446.42 ± 21.89, microglia conditioned medium: 465.35 ± 24.55, *p* = 0.474). As previously described, colocalizations of both signals indicate structural inhibitory synapses (Figures 8E–G). Here, neurons treated with conditioned medium of activated microglia formed significantly less colocalizations (30.02 ± 2.22; *p* = 0.0006) than neurons of the control condition (36.93 ± 1.62) (Figure 8J).

**Figure 9:**
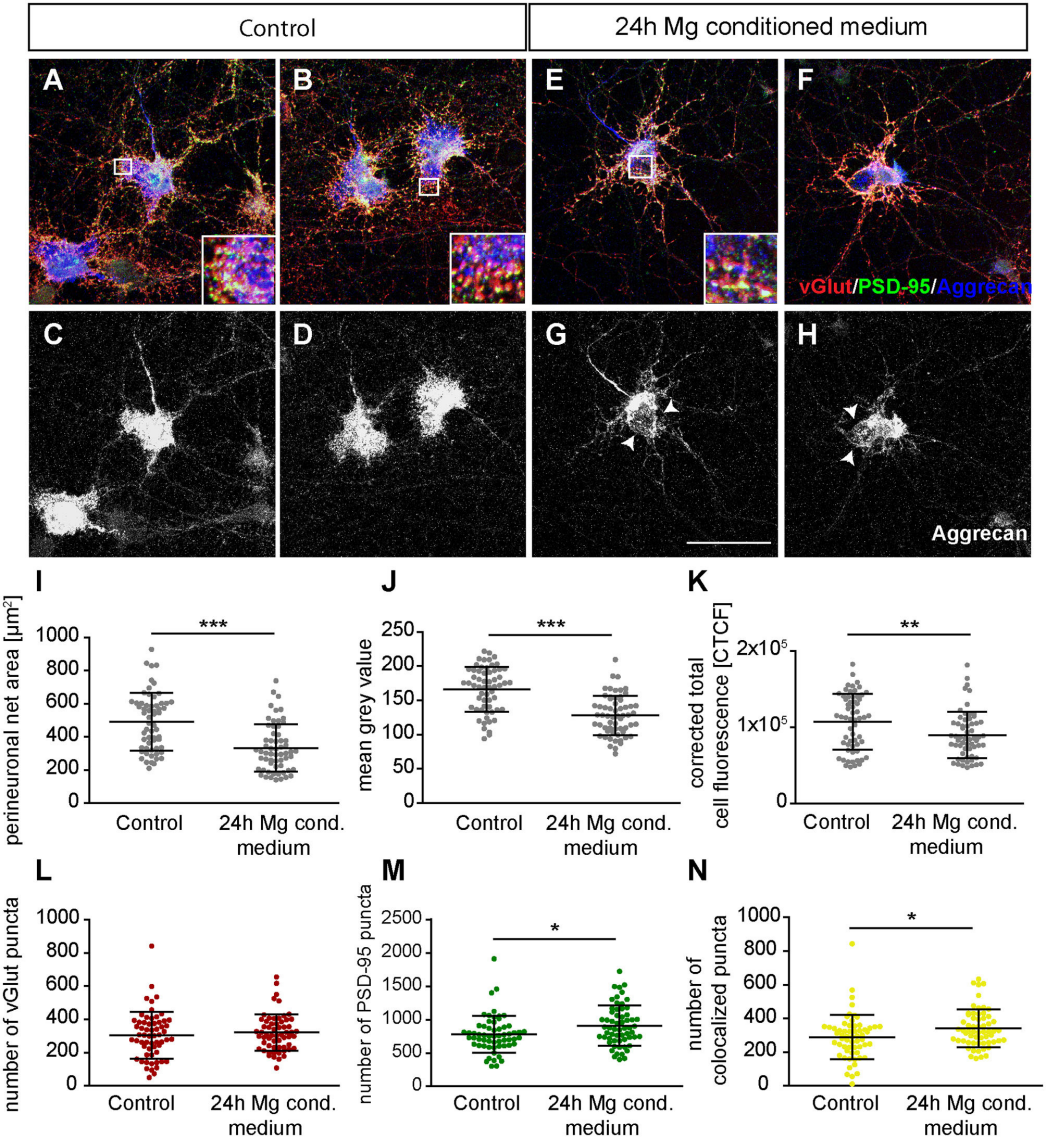
**(A,B)** Representative confocal laser scanning microscopy images of structural glutamatergic synapses on perineuronal net wearing neurons after a treatment with fresh hippocampus medium (controls). Neurons were immunocytochemically stained against vGlut (red), PSD-95 (green), and Aggrecan (blue), a major PNN component; **(C,D)** Grayscale images of Aggrecan positive PNNs. PNNs were evenly covering the soma and proximal dendrites of cultured hippocampal neurons (scale bar: 100 μm); **(E–H)** Representative immunostainings of excitatory synapses on PNN positive neurons after a 24 h treatment with conditioned medium of activated microglia. Grayscale depictions show a clear disruption of Aggrecan containing PNNs as indicated by arrows; **(I–K)** Quantification of PNN parameters showed a significant reduction in PNN area and staining intensity; **(L–N)** Analysis of synaptic puncta showed no changes with regard to presynaptic vGlut signals. However, a significantly raised number of postsynaptic PSD-95 and colocalized puncta could be verified. Statistics: Three independent experimental repetitions (*N* = 3) were performed for the analysis of synaptic puncta PNN parameters. 20 neurons (*n* = 20) were recorded and quantified per repetition. Data are shown as mean ± SD (*t*-test, *p ≤ 0.05, **p ≤ 0.01, and ***p ≤ 0.001). The data dots in **(I–N)** represent individual values of single neurons.

Next, we focused on the analysis of the PNN-wearing neuronal population which was identified by the presence of an Aggrecan signal. Here, the signal for Aggrecan was used to analyze PNN parameters after the treatment with microglia conditioned medium (Figures 9A–H). Neurons of the control group formed dense PNNs covering evenly the soma and proximal dendrites (Figures 9A–D). In contrast, neurons treated for 24 h with microglia conditioned medium showed an astonishing disruption of PNNs (Figures 9E–H, indicated by arrows). The quantification of PNN areas revealed that neurons of the control group formed PNNs with an average area of 492.01 ± 22.44 μm^2^ while treated neurons formed PNNs with a significantly smaller area of 333.94 ± 18.57 μm^2^ (*p* < 0.0001) (Figure 9I). We also analyzed the fluorescence intensity by determining the mean gray value and corrected total cell fluorescence (CTCF) (Figures 9J,K). In the control group a mean gray value of 166.18 ± 4.25 was observed. In contrast, treated neurons showed a significantly weaker mean gray value of 128.04 ± 3,74 (*p* < 0.0001). The CTCF value was also significantly reduced in the microglia conditioned medium treatment group (89999.10 ± 3934.90; *p* = 0.0061) compared to the control situation (107204.23 ± 4744.47).

**Figure 10:**
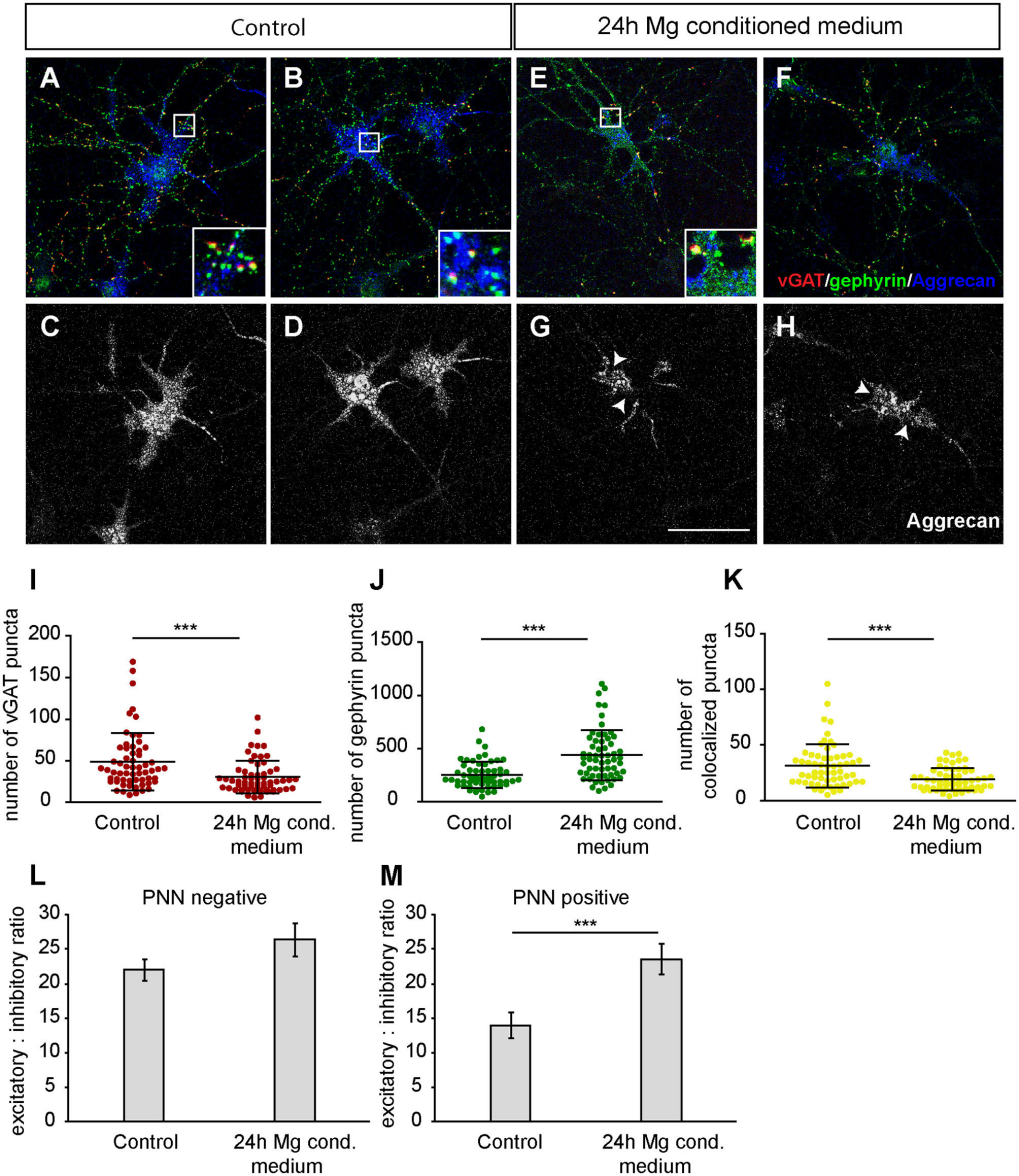
**(A,B)** Representative confocal laser scanning microscopy images of structural GABAergic synapses on perineuronal net wearing neurons after a 24 h incubation with fresh hippocampus medium (controls). Neurons were immunostained with antibodies against vGAT (red), gephyrin (green) and Aggrecan (blue), which can be found in all mature PNNs; **(C,D)** Grayscale images of Aggrecan positive PNNs that were evenly covering the soma and proximal dendrites of cultured neurons (scale bar: 100 μm); **(E–H)** Representative recordings of inhibitory synapses on PNN positive neurons after a 24 h treatment with conditioned medium of Poly I:C activated microglia. Grayscale depictions show a clear disruption of Aggrecan containing PNNs as indicated by arrows; **(I)** Quantification of presynaptic vGAT puncta revealed a significant reduction when neuronal networks were treated with microglia conditioned medium; **(J)** In contrast, postsynaptic gephyrin puncta were significantly increased on neurons with disrupted perineuronal nets; **(K)** Number of structural inhibitory synapses was significantly decreased after the treatment with conditioned medium of activated microglia; **(L)** For the analysis of excitatory and inhibitory balance, a ratio was formed between the colocalized excitatory and colocalized inhibitory fluorescence of PNN negative neurons. Here, no significant alteration could be observed in the excitatory: inhibitory ratio; **(M)** The excitatory: inhibitory ratio was also determined for PNN wearing neurons and showed a significant increase when neurons were treated for 24 h with microglia conditioned medium of activated microglia. This indicates a shift toward a higher excitatory input compared to control neurons treated with the freshly prepared medium. Statistics: Three independent experimental repetitions (*N* = 3) were performed for the analysis of synaptic puncta parameters and ratio formation. 20 neurons (*n* = 20) were recorded and quantified per repetition. Data of puncta analysis are shown as mean ± SD (Mann-Whitney *U*-test, *p ≤ 0.05, **p ≤ 0.01 and ***p ≤ 0.001) and data of ratio analysis are shown as mean ± SEM (Mann-Whitney U-test, *p* ≤ 0.05). Single data dots in **(I–K)** are representing puncta numbers of single neurons.

Furthermore, we focused on glutamatergic and GABAergic synapse numbers on PNN wearing neurons (Figures 9, 10). Here, no significant alterations could be observed for vGlut puncta (Control: 304.88 ± 18.13, microglia conditioned medium: 321.18 ±14.10; *p* = 0.479) (Figure 9L). However, a mild but significantly increased number of PSD-95 puncta was observed when neurons were treated for 24 h with microglia conditioned medium (908.75 ± 38.80; *p* = 0.0157) in comparison to control neurons (779.98 ± 35.37) (Figure 9M). The number of colocalized glutamatergic signals was also significantly increased in the treated condition (341.62 ± 14.38; *p* = 0.0216) compared to the control condition (289.67 ± 17.07) (Figure 9N). Interestingly, a prominent and significant reduction of presynaptic vGAT puncta was visible when neurons were treated with microglia conditioned medium (30.55 ± 2.53; *p* = 0.0003) in comparison to the control condition (48.82 ± 4.45) (Figure 10I). Neurons treated with microglia conditioned medium formed significantly more postsynaptic gephyrin signals (439.85 ± 30.46; *p* < 0.0001) than control neurons (252.23 ± 15.88) (Figure 10J). Nevertheless, the number of colocalized inhibitory signals was significantly lower when neurons were treated with microglia conditioned medium (Control: 19.03 ± 1.3, microglia conditioned medium: 31.2 ± 2.52; *p* < 0.0001). Finally, the ratio between structural excitatory and inhibitory synapses was calculated for PNN wearing and PNN negative neurons to analyze if a shift in synaptic balanced occurred after the administration of microglia conditioned medium (Figures 10L,M). For PNN negative neurons a ratio of 22.06 ± 1.52 excitatory structural synapses to 1 structural inhibitory synapse could be observed in the control group. In comparison, neurons treated with the conditioned medium of activated microglia showed a ratio of 26.41 ± 2.43 excitatory synapse to 1 inhibitory synapse which not significantly changed compared to the control (Figure 10L; *p* = 0.1375). PNN wearing neurons of the control group formed a ratio of 14.04 ± 1.83 excitatory synapses to 1 inhibitory synapse. Interestingly, PNN wearing neurons treated for 24 h with the conditioned medium of activated microglia showed a significant (*p* < 0.0001) shift to a ratio of 23.60 ± 2.21 excitatory synapse to 1 inhibitory synapse, indicating a higher excitatory input on the PNN wearing subgroup of neurons in the culture (Figure 10M).

## Discussion

In our study, we focused on the identification of microglial secreted factors and their impact on cultured neuronal networks. Primary microglia showed a typical *in vitro*- morphology as previously described (Rustenhoven et al., 2016; Zhao et al., 2018; Haage et al., 2019). The treatment of microglia with increasing concentrations of Poly I:C induced a rise in the number of completely rounded, amoeboid-shaped microglia (Figure 1C). This morphological change was extensively described by Town and colleagues who showed that microglia react through TLR-3 signaling to Poly I:C treatment (Town et al., 2006). While several publications describe the induced apoptosis of neurons by activated microglia (Field et al., 2010), less is known about microglial apoptosis induced by high concentrations of Poly I:C. Here, we could observe a significantly increased number of apoptotic microglia when an incubation with 100 μg/ml Poly I:C was performed. A similar observation was made when microglia were treated with increasing concentrations of lipopolysaccharide (LPS) *in vitro* (Jung et al., 2005). Jung et al. (2005) identified the NF-κB downstream activation and the release of interferon- β (IFN-β) as a possible mechanism for the induced apoptosis of microglia. When microglia were treated with 50 μg/ml Poly I:C for 24 h, the existence of different cytokines and MMPs could be observed via RT-PCR or cytokine array analysis (Figure 2). A similar pattern of factors in a cytokine array analysis was observed when primary microglia were activated through LPS or the ECM glycoprotein tenascin-c (TN-C), supporting the activated state of microglia in our cultures (Haage et al., 2019). Unfortunately, cultured microglia showed a quite high activity state in the untreated vehicle control (0 μg/ml Poly I:C) which was also observed with regard to cytokine levels (Supplementary Figures 1A,B). We assume that this level of activity was caused by primary culturing procedures and the presence of apoptotic microglia in the cultures (Figure 1D). This observation was supported by electrophysiological MEA recordings which showed a similar effect on spike number increase per electrode when microglia conditioned medium without Poly I:C was added to neurons (Supplementary Figure 1C). In addition, it is noteworthy that the baseline recording of neurons treated with microglia conditioned medium without Poly I:C was significantly lower compared to the other conditions (Supplementary Figure 1D). A direct comparison between inactive and active microglia was for these reasons not possible and excluded for further analysis. However, the analysis of inactive microglia and their released factors is of great interest and might be performed preferably using microglia or microglia-like cell lines in future studies.

Interestingly, it is known that some of the identified cytokines have a strong stimulating effect on neuronal activity, as observed in our MEA measurements. The C-X-C motif ligand family members CXCL-1 and CXCL-2 showed the highest levels in the supernatant of stimulated microglia (Figures 2D,E,G). Both are ligands of the CXCR2 receptor, which is expressed in the murine and human hippocampus (Hesselgesser and Horuk, 1999; Xu et al., 2017). For CXCL-1 it has been shown that its binding to CXCR2 induces an upregulation of Na^+^ currents and an increase of the excitability of isolated small diameter sensory neurons as well as in cerebellar slices with a participation of the MEK/ERK pathway (Ragozzino et al., 1998; Wang et al., 2008). Less is known about the influence of CXCL-2 on neuronal excitability. Since CXCL-2 shares the same receptor as CXCL-1 it might also possess a stimulatory effect on excitability. In contrast, CXCL-10 acts through CXCR3 receptor signaling. For cultured hippocampal neurons it has been described that CXCL-10 rises the intracellular Ca^2+^ levels and enhances the spontaneous and evoked electrical activity (Nelson and Gruol, 2004; Cho et al., 2009). In addition, Nelson and colleagues verified the presence of the CXCR3 receptor on the surface of cultured neurons. For CXCL-12, its influence on synaptic release of glutamate and GABA has extensively been described (Guyon, 2014). However, CXCL-12 was detected only in exceptionally low concentrations in the microglia-conditioned supernatant in our experiments.

Another chemokine ligand family found in the microglia conditioned medium was the C-C motif ligand family (Figures 2D–F). Here, CCL5 was observed in exceedingly high levels. For primary hippocampal neurons the expression of the CCL5 receptors CCR1 and CCR5 could be verified on mRNA level (Meucci et al., 1998). In human synaptosomes CCR1, CCR3, and CCR5 could be verified also on protein level (Musante et al., 2008). Treatment of human and murine synaptosomes with CCL5 facilitated the spontaneous release of glutamate by the recruitment of Ca^2+^ from inositol trisphosphate-sensitive stores (IP3) and cytosolic tyrosine kinase-mediated phosphorylations (Musante et al., 2008). Additionally, a previously published study demonstrated that CCL2, which is binding to the corresponding CCR2 receptor, increased spike firing in *cornu ammonis* (CA) neuronal cells and produced an increase of excitatory postsynaptic currents (EPSCs) in Schaffer-collateral fibers in rat hippocampal slices (Zhou et al., 2011). Similar results have been observed for cultured hippocampal neurons treated with CCL3 which binds to CCR1, CCR3, and CCR5 (Baba and Mukaida, 2014). Here, an increase of Ca^2+^ signals accompanied with a higher spontaneous network activity was observed when cultures were treated with CCL3 (Kuijpers et al., 2010). Little is known about the impact of CCL4 on neuronal activity, however CCL4 binds to CCR5 and might therefore unfold a similar effect on neuronal activity, as previously mentioned (Samson et al., 1996; Musante et al., 2008).

TNF-α and IL-6 were also detected in the supernatant of activated microglia. It is known that hippocampal neurons express TNF-R1 and TNF-R2 receptors on their cell surface (Neumann et al., 2002). The analysis of TNF-α isolated from kainic acid activated microglia revealed an increase of voltage-gated Ca^2+^-currents in hippocampal neurons (Zhu et al., 2010). Interestingly, these observations were accompanied by an upregulation of N-methyl-D-aspartate receptor-1 (NMDAR1) and inducible nitric oxide synthases (iNOS) expression (Zhu et al., 2010). Similar results have been also obtained for Ca^2+^ permeable α-amino-3-hydroxy-5-methyl-4-isoxazolepropionic acid receptors (AMPAR) *in vivo* and *in vitro* (Beattie et al., 2002; Ogoshi et al., 2005). The activation of TNF-R1 and TNF-R2 induces the TNF receptor associated factor-2 (TRAF2) downstream signaling and the activation of caspases and ceramide (Pickering et al., 2005). Therefore, TNF-α is suspected to be an important inducer of excitotoxicity. Contrarily, inhibitory effects have been shown for IL-6 on synaptic transmission. The acute treatment of isolated cortical synaptosomes with IL-6 revealed an inhibition of glutamate release (D'Arcangelo et al., 2000). Field excitatory post-synaptic potential recordings of the CA1 region in hippocampal slices after IL-6 administration showed a reduction in LTP and STP, while basal neurotransmission was not affected (Li et al., 1997). Moreover, dissociated hippocampal neurons expressed IL6Rα receptors and developed decreased Ca^2+^ levels when a chronic treatment with IL-6 was performed (Vereyken et al., 2007). IL-6 was the only detected cytokine with inhibitory effects on synaptic transmission. However, we suppose that its effect might simply be overruled by all other, mainly stimulating acting cytokines. There are no evidences for GM-CSF and G-CSF with regard to neuronal excitability however they unfold strong neuroprotective and anti-apoptotic properties (Schneider et al., 2005; Nishio et al., 2007; Choi et al., 2011; Kelso et al., 2015).

The conditioned medium of activated microglia led to a strong disruption of PNNs when neurons were treated for 24 h (Figures 9, 10). Since we could observe the expression of MMPs by activated microglia, we assume that the impaired PNN integrity might be caused by their protease activity. Previous studies revealed that activated microglia are a source of MMP-2 and MMP-9 (Könnecke and Bechmann, 2013). Especially, MMP-9 is often co-localized with NMDA and AMPA receptors as well as with vGlut and therefore supposed to be involved in synaptic plasticity (Gawlak et al., 2009; Huntley, 2012). Interestingly, it has been shown that MMP-2 and MMP-9 can unfold their enzymatic activity on Aggrecan, Brevican and Neurocan, which can be found in PNNs (Overall, 2002; Ethell and Ethell, 2007). Aggrecan is a core component of PNNs in the CNS (Giamanco et al., 2010) and a disruption might therefore explain our observations. Furthermore, microglia are also a major source of a disintegrin and metalloproteinase with thrombospondin motifs (ADAMTS) (Tauchi et al., 2012a,b). For this protease family, a more efficient degradation of Aggrecan was described in comparison to MMPs (Durigova et al., 2011). Therefore, members of the ADAMTS protease family might also contribute to the degradation of PNNs in our experiments. Another explanation for the disrupted PNNs could be the induction of neuronal apoptosis by toxic hyperexcitability indicated in the MEA measurements.

Alterations or disruptions of PNNs can alter the synaptic plasticity and the excitatory/inhibitory balance (Gottschling et al., 2019). Therefore, we analyzed glutamatergic and GABAergic synapse numbers on PNN wearing neurons. Concomitant with the disrupted PNN structure, both postsynaptic markers, PSD-95 and gephyrin were significantly increased (Figures 9M, 10J). In contrast, the same postsynaptic proteins remained unchanged on PNN negative neurons (Figures 7I, 8I). PNNs are discussed to act as physical barriers that inhibit synapse formation and reduce synaptic plasticity (Wang and Fawcett, 2012; Sorg et al., 2016). In addition, an interesting study by Frischknecht and colleagues revealed that PNNs decrease lateral receptor motility and the exchange efficiency of receptors from extra-synaptic reservoirs (Frischknecht et al., 2009). Therefore, a disruption of PNNs could enhance the recruitment of postsynaptic scaffold proteins for the formation of new synapses. This assumption is supported by a previous study which revealed that an enzymatic digestion of PNNs by Chondroitinase ABC (ChABC) or hyaluronidase increased the number of postsynaptic Shank2 and ProSAP1 puncta (Pyka et al., 2011). Presynaptic vGlut proteins were unaffected by the disruption of PNNs, while the number of inhibitory vGAT puncta was significantly reduced. This observation indicates a differential impact of PNNs on excitatory and inhibitory presynaptic terminals. A digestion of PNNs by ChABC and hyaluronidase did not change presynaptic Bassoon signals but preferentially proteins of the postsynaptic density (Pyka et al., 2011). A recently published study revealed that a lack of the four PNN components Tenascin-c, Tenascin-r, Brevican, and Neurocan altered the ratio of excitatory and inhibitory synapses with an increase of glutamatergic and a decrease of GABAergic synapse numbers (Gottschling et al., 2019). This data also supports the observation of increased glutamatergic colocalizations and decreased GABAergic colocalizations on neurons with disrupted PNNs in the present study. However, the reduction of presynaptic vGAT puncta might be also explained by the loss of GABAergic interneurons which are more sensitive and represented in a lower number than excitatory pyramidal neurons. A mild but significant reduction of both presynaptic proteins was observed on PNN negative neurons, which might be induced by repulsive acting cytokines. Especially for TNF- α and members of the CXCL-motif cytokine family inhibitory effects on axon and neurite outgrowth have been described (Neumann et al., 2002; Deftu et al., 2019). Since the treatment of hippocampal neurons occurred at an early phase of synapse formation (DIV12) this could be a possible explanation for the reduced number of vGlut and vGAT puncta on PNN negative neurons.

The increased neuronal network activity might appear surprising with regard to the overall reduction of structural synapse numbers on PNN negative neurons and the increased number of glutamatergic synapses on PNN wearing neurons. In addition, the ratio analysis of structural synapses revealed a shift to a higher number of excitatory synapses in relation to inhibitory synapses on neurons with PNN disturbances induced by the treatment with microglia conditioned medium (Figure 10M). PNNs are often associated with fast-spiking parvalbumin positive interneurons, as already mentioned in the introduction (Brückner et al., 1994; Härtig et al., 1994; Celio et al., 1998; Carulli et al., 2010; Fawcett et al., 2019). Interestingly, in a previous study it has been observed that a degradation of PNNs via ChABC treatment increased the excitability of interneurons (Dityatev et al., 2007). In a simple model, the increased number of excitatory inputs on PNN positive neurons might therefore enhance the regional release of GABA and rather reduce the network activity in a region-specific manner. To analyze if specific subpopulations of neurons (e.g., fast spiking interneurons) react with a higher or lower spike and burst firing frequency after the administration of microglia conditioned medium, we created plots where the baseline frequency of every single electrode was juxtaposed against the frequency 3, 24, and 48 h post-treatment (Figures 5A–I, 6A–I). However, the activity increase did not depend on neurons with a certain baseline frequency. This shows rather an overall reaction of the neuronal network on treatment. Interestingly, similar observations were made in the previously mentioned quadruple knockout mouse model lacking the four PNN components tenascin-C, tenascin-R, Brevican, and Neurocan (Gottschling et al., 2019). Here, an increase of the spontaneous network activity was observed via MEA analysis accompanied by an increase of glutamatergic and a decrease of GABAergic synapse numbers on PNN wearing neurons. In another study, a complete digestion of perineuronal nets with hyaluronidase increased the spike and burst firing rate of cultured neurons on MEAs (Bikbaev et al., 2015). It is necessary to consider that PNNs also restrict lateral diffusion of neurotransmitter receptors (Frischknecht et al., 2009) and might furthermore prevent the diffusion of neurotransmitter from the synaptic cleft, as discussed by Gundelfinger et al. (2010). Because of this multifunctional role of PNNs, it is difficult to speculate about the firing behavior of PNN wearing neurons and its effect on the whole network activity without performing a specific functional analysis of this subtype. Future studies might utilize interneuron specific calcium imaging via AAV transduction and analyze the firing behavior of this subpopulation more specifically.

Neuronal apoptosis might be another possible reason for the increased activity on MEAs. As above mentioned, previous studies showed that microglia can induce the apoptosis of neurons (Field et al., 2010). To exclude that apoptosis and the loss of larger network regions are responsible for the activity increase, we controlled the percentage of active electrodes (Figures 5J–L). Here, we could observe a constant percentage of active electrodes above 90% for all conditions. This observation does not indicate a widespread cell death in our cultures. The last aspect that should be discussed, is that neuronal networks of all conditions showed an increase in spike and burst numbers 3 h after the addition of medium, Poly I:C containing medium or microglia conditioned medium. We assume that this increase was induced by the partially performed 25 % medium exchange and the consequent dilution of astrocytic factors which were previously secreted into the commonly shared culture medium of neurons and astrocytes. The activity increase sustained only in the microglia conditioned medium group for 24 and 48 h.

In summary, we could show that microglial secreted factors disrupt perineuronal nets and alter the number of glutamatergic and GABAergic synapses depending on the presence of PNNs. These alterations are also accompanied by an increased electrophysiological spontaneous activity.

## Data Availability Statement

The raw data supporting the conclusions of this article will be made available by the authors, without undue reservation.

## Ethics Statement

The animal study was reviewed and approved by Landesamt für Umweltschutz, Naturschutz und Verbraucherschutz, North Rhine-Westphalia, D-45659 Recklinghausen, Germany.

## Author Contributions

AF and GJ designed and supervised the study. DW performed the experiments, quantified, and interpreted the data. AF, GJ, and NF interpreted the data. DW drafted the manuscript. AF, GJ, and NF revised the manuscript. All authors contributed to the article and approved the submitted version.

## Conflict of Interest

The authors declare that the research was conducted in the absence of any commercial or financial relationships that could be construed as a potential conflict of interest.

## Supplementary Material

Supplementary Video 1 Primary cortical microglia were cultured on a PDL-coating for 2 days *in vitro* and then recorded via video microscopy for 72 h. Microglia showed a typical morphology and monitored their surroundings. In addition, phagocytosis events could be observed, which are indicated by red dots in the recording.

Supplementary Figure 1 **(A,B)** Cytokine array of microglia conditioned medium without the administration of Poly I:C. The cytokine levels in the supernatant of cultured microglia without Poly I:C were similar to the previously determined cytokine levels in the supernatant of Poly I:C activated microglia. This supports the observation of a certain microglia activity level in untreated cultures (Figure 1C); **(C)** When the conditioned medium of untreated microglia (light blue) was added to cultured hippocampal neurons on MEAs, a similar increase of spikes per electrode was observed compared to neurons treated with the conditioned medium of activated microglia (dark blue). However, the conditioned medium of activated microglia showed a significant increase of spikes after 3, 24, and 48 h compared to the vehicle control; **(D)** Comparison of baselines between all groups revealed a significantly lowered number of spikes in the microglia conditioned medium group without Poly I:C compared to all other groups. For reasons of comparability this data set was not considered for further analysis. Statistics: Three independently collected samples (*N* = 3) were used for the analysis of cytokine levels in the supernatant of Poly I:C activated microglia and one (*N* = 1) sample was used for the analysis of cytokine levels in the supernatant of microglia conditioned medium without Poly I:C. For the MEA analysis five independent experiments (*N* = 5) were performed for microglia conditioned medium without Poly I:C and data of four independent experiments were used for microglia conditioned medium with Poly I:C. In total, data of 240–300 electrodes were considered for MEA quantification. Data in C and D are shown as mean ± SD (pairwise comparison via Mann-Whitney *U*-test, *p* ≤ 0.05).
