# Supplementary figures and images for: Poly I:C Activated Microglia Disrupt Perineuronal Nets and Modulate Synaptic Balance in Primary Hippocampal Neurons in vitro
Source: Front Synaptic Neurosci. 2021 Feb 23;13:637549. doi: 10.3389/fnsyn.2021.637549 (PMC7940526; doi:10.3389/fnsyn.2021.637549)

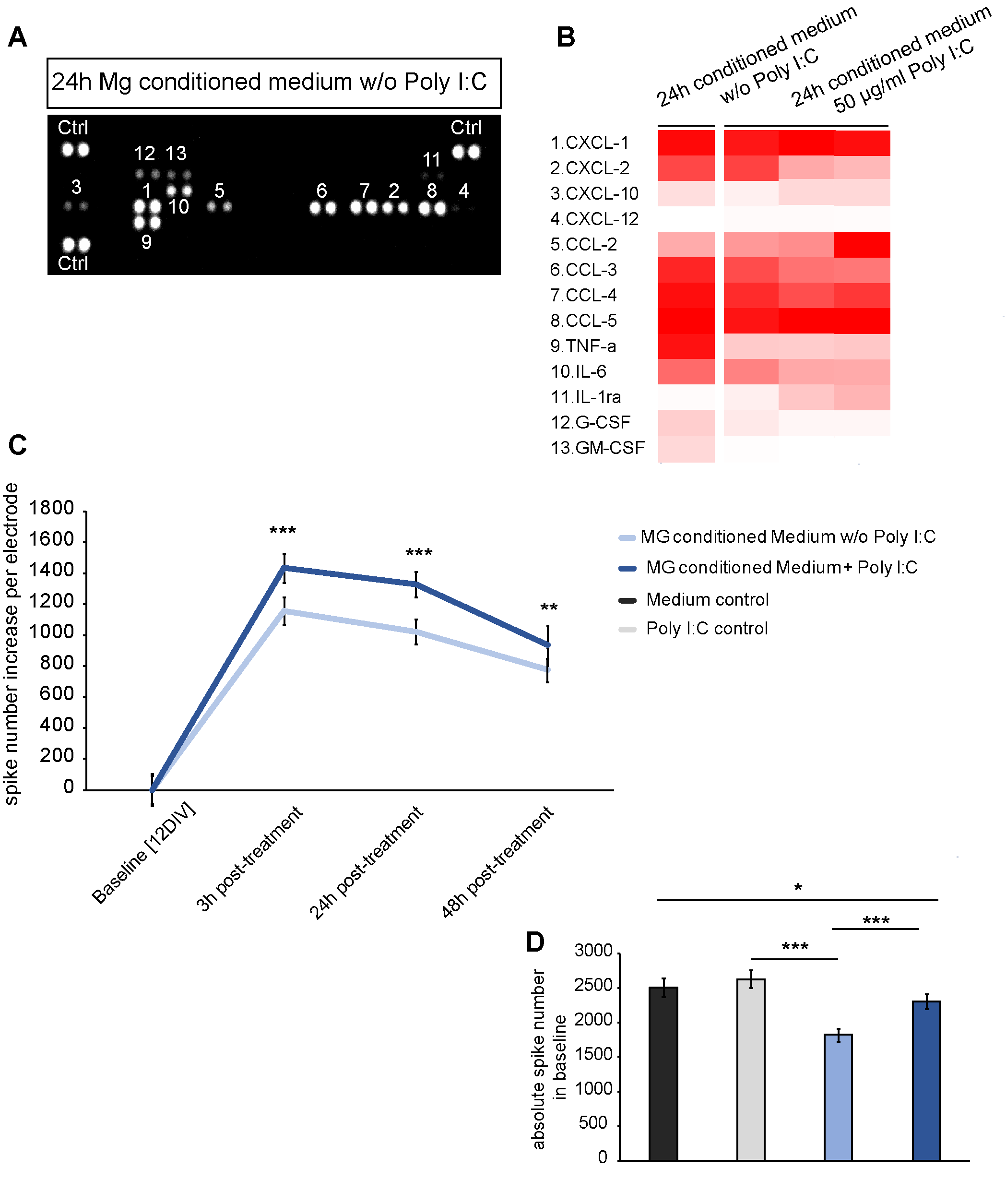

Supplement: Supplementary Figure 1 — (A,B) Cytokine array of microglia conditioned medium without the administration of Poly I:C. The cytokine levels in the supernatant of cultured microglia without Poly I:C were similar to the previously determined cytokine levels in the supernatant of Poly I:C activated microglia. This supports the observation of a certain microglia activity level in untreated cultures (Figure 1C); (C) When the conditioned medium of untreated microglia (light blue) was added to cultured hippocampal neurons on MEAs, a similar increase of spikes per electrode was observed compared to neurons treated with the conditioned medium of activated microglia (dark blue). However, the conditioned medium of activated microglia showed a significant increase of spikes after 3, 24, and 48 h compared to the vehicle control; (D) Comparison of baselines between all groups revealed a significantly lowered number of spikes in the microglia conditioned medium group without Poly I:C compared to all other groups. For reasons of comparability this data set was not considered for further analysis. Statistics: Three independently collected samples (N = 3) were used for the analysis of cytokine levels in the supernatant of Poly I:C activated microglia and one (N = 1) sample was used for the analysis of cytokine levels in the supernatant of microglia conditioned medium without Poly I:C. For the MEA analysis five independent experiments (N = 5) were performed for microglia conditioned medium without Poly I:C and data of four independent experiments were used for microglia conditioned medium with Poly I:C. In total, data of 240–300 electrodes were considered for MEA quantification. Data in C and D are shown as mean ± SD (pairwise comparison via Mann-Whitney U-test, p ≤ 0.05). [file Image_1.TIF]
